# On the relationship between cloud water composition and cloud droplet number concentration

**DOI:** 10.5194/acp-20-7645-2020

**Published:** 2020-07-02

**Authors:** Alexander B. MacDonald, Ali Hossein Mardi, Hossein Dadashazar, Mojtaba Azadi Aghdam, Ewan Crosbie, Haflidi H. Jonsson, Richard C. Flagan, John H. Seinfeld, Armin Sorooshian

**Affiliations:** 1 Department of Chemical and Environmental Engineering, University of Arizona, Tucson, AZ, USA; 2 Science Systems and Applications, Inc., Hampton, VA, USA; 3 NASA Langley Research Center, Hampton, VA, USA; 4 Naval Postgraduate School, Monterey, CA, USA; 5 Department of Chemical Engineering, California Institute of Technology, Pasadena, CA, USA; 6 Department of Hydrology and Atmospheric Sciences, University of Arizona, Tucson, AZ, USA

## Abstract

Aerosol-cloud interactions are the largest source of uncertainty in quantifying anthropogenic radiative forcing. The large uncertainty is, in part, due to the difficulty of predicting cloud microphysical parameters, such as the cloud droplet number concentration (*N*_d_). Even though rigorous first-principle approaches exist to calculate *N*_d_, the cloud and aerosol research community also relies on empirical approaches such as relating *N*_d_ to aerosol mass concentration. Here we analyze relationships between *N*_d_ and cloud water chemical composition, in addition to the effect of environmental factors on the degree of the relationships. Warm, marine, stratocumulus clouds off the California coast were sampled throughout four summer campaigns between 2011 and 2016. A total of 385 cloud water samples were collected and analyzed for 80 chemical species. Single- and multispecies log-log linear regressions were performed to predict *N*_d_ using chemical composition. Single-species regressions reveal that the species that best predicts *N*_d_ is total sulfate ($R_{\text{adj}}^{2}=0.40$). Multispecies regressions reveal that adding more species does not necessarily produce a better model, as six or more species yield regressions that are statistically insignificant. A commonality among the multispecies regressions that produce the highest correlation with *N*_d_ was that most included sulfate (either total or non-sea-salt), an ocean emissions tracer (such as sodium), and an organic tracer (such as oxalate). Binning the data according to turbulence, smoke influence, and in-cloud height allowed for examination of the effect of these environmental factors on the composition-*N*_d_ correlation. Accounting for turbulence, quantified as the standard deviation of vertical wind speed, showed that the correlation between *N*_d_ with both total sulfate and sodium increased at higher turbulence conditions, consistent with turbulence promoting the mixing between ocean surface and cloud base. Considering the influence of smoke significantly improved the correlation with *N*_d_ for two biomass burning tracer species in the study region, specifically oxalate and iron. When binning by in-cloud height, non-sea-salt sulfate and sodium correlated best with *N*_d_ at cloud top, whereas iron and oxalate correlated best with *N*_d_ at cloud base.

## 1 Introduction

To assess the degree to which humans have altered Earth’s climate, it is necessary to quantify the effect that particles in the air (i.e., aerosols) have on clouds. Some fraction of aerosols (called cloud condensation nuclei, CCN) activate into cloud droplets, thus impacting the cloud droplet number concentration (*N*_d_). For warm marine boundary layer (MBL) clouds at fixed liquid water, higher *N*_d_ values result in (i) higher cloud albedo (thus cooling the Earth and counteracting the greenhouse effect) (Twomey, 1977), (ii) delayed and/or reduced precipitation (Albrecht, 1989), and (iii) enhanced entrainment at cloud top (Ackerman et al., 2004). The complex interactions and feedback mechanisms between aerosols, meteorology, and clouds leads to aerosol-cloud interactions as the largest source of uncertainty in climate models (IPCC, 2013; Bellouin et al., 2020).

It is indispensable to know the value of *N*_d_, but this is a difficult parameter to accurately simulate and retrieve (Fountoukis and Nenes, 2005). There is a need to improve *N*_d_ retrievals from satellite remote sensors, which provide broad spatial and temporal coverage in contrast to surface sites and airborne research flights. Currently, *N*_d_ retrievals are limited to inferred values based on values of cloud optical depth, cloud droplet effective radius, and temperature, along with assumptions such as vertical homogeneity of *N*_d_ and monotonic increases in liquid water content at a constant fraction of its adiabatic value (Grosvenor et al., 2018). Ultimately, measurements are needed to better inform climate models about the cloud droplet activation process and better constrain *N*_d_ values. Current general circulation models (GCMs) calculate *N*_d_ using the properties of aerosol particles in one of two ways (Ghan et al., 1997; Menon et al., 2002). First, there is a rigorous approach that is based on physical principles that predicts *N*_d_ based on aerosol properties and meteorological conditions (Abdul-Razzak and Ghan, 2000). Second, there is an empirical approach that parameterizes *N*_d_ using either the number concentration of aerosols, *N*_a_ (cm^−3^); the number concentration of CCN, *N*_ccn_ (cm^−3^); or the mass concentration of chemical species that comprise the aerosols (Ghan et al., 1997).

The rigorous approach predicts *N*_d_ by considering aerosol properties (e.g., size distribution and chemical composition), microphysical processes (e.g., the seeding of cloud droplets by particles, droplet growth, and droplet evaporation), and meteorological parameters (e.g., relative humidity and the vertical updraft velocity transporting aerosols to cloud base) (e.g., Chuang et al., 1992; Chuang and Penner, 1995; Nenes and Seinfeld, 2003; Partridge et al., 2012). This method is based on the physical principle that an aerosol particle needs to be a cloud condensation nucleus in order to seed a cloud droplet; consequently, the input for this approach is *N*_a_, from which to calculate *N*_ccn_, and subsequently *N*_d_. The requisite information for these calculations may not be readily available for GCMs. A limitation is that the spatial resolution of a GCM may be too coarse to capture the small-scale spatial variation of updraft velocity (Ghan et al., 2011; West et al., 2014).

The empirical parameterization approach of interest in the present study uses the mass concentration of one or several chemical species and correlates it directly to *N*_ccn_ or *N*_d_. Aerosols containing the sulfate ion (${\text{SO}}_{4}^{2-}$) have long been known to serve as effective CCN (Andreae and Rosenfeld, 2008; Charlson et al., 1992; Lance et al., 2009; Medina et al., 2007). Sulfate is both contained in sea salt and is a product of the oxidation of gaseous sulfur dioxide (SO_2_) (Hegg et al., 1981; Quinn et al., 2017), so it is customary to isolate the anthropogenic contribution to total ${\text{SO}}_{4}^{2-}$ by considering its non-sea-salt fraction (${\text{NSS-SO}}_{4}^{2-}$). Therefore, most studies choose either total ${\text{SO}}_{4}^{2-}$ (denoted hereafter as ${\text{Tot-SO}}_{4}^{2-}$) or ${\text{NSS-SO}}_{4}^{2-}$ to predict *N*_ccn_ and *N*_d_ (e.g., Leaitch et al., 1992; Novakov et al., 1994; Saxena and Menon, 1999). Using the mass concentration of ${\text{SO}}_{4}^{2-}$ or any other chemical species to predict *N*_d_ (i) circumvents the complex intermediate microphysical steps to go from an aerosol particle to a cloud droplet and implicitly accounts for such meteorological variables like updraft velocity, (ii) is based on actual measurements, and (iii) can be compared directly to the mass concentration of different species produced by aerosol transport models (e.g., Boucher and Lohmann, 1995; Chen and Penner, 2005). The limitations of using an empirical parameterization are (i) assuming a mass size distribution of the aerosols, (ii) assuming that one or a few chemical species are responsible for all CCN, and (iii) uncertainty in generalizing field data from one region (or a few regions) under specific conditions to the entire globe for all conditions (Pringle et al., 2009). Despite these drawbacks, empirical correlations of *N*_d_ and the mass concentration of different species are valuable. For example, of the 20 studies addressing the cloud albedo effect considered in the IPCC Fourth Assessment Report (IPCC, 2007), half relied on empirical relationships to calculate *N*_d_ (Pringle et al., 2009).

Several studies have developed empirical correlations between *N*_CCN_ and the mass concentration of ${\text{SO}}_{4}^{2-}$ (e.g., Adams and Seinfeld, 2003; Hegg et al., 1993; Matsumoto et al., 1997). However, the present objective is to focus on improving the prediction of *N*_d_, not *N*_CCN_, using the mass concentration of ${\text{SO}}_{4}^{2-}$ in addition to other species. A log-log relation is often used to correlate the mass concentration of ${\text{SO}}_{4}^{2-}$ to *N*_d_ with an equation of the following form (e.g., Lowenthal et al., 2004):

(1) $$\text{log}\left(N_{\text{d}}\right)=a_{0}+a_{1}\text{ log}\left(\left[{\text{SO}}_{4}^{2-}\right]\right),$$

where ${\text{SO}}_{4}^{2-}$ is the mass concentration in air (μg m^−3^), and *a*_0_ and *a*_1_ are fitting parameters. A log-log relation is chosen to accommodate large ranges in *N*_d_ and ${\text{SO}}_{4}^{2-}$ and to reduce sensitivity of results to the measurement accuracy of each individual parameter (Boucher and Lohmann, 1995). The mass concentration of ${\text{SO}}_{4}^{2-}$ can be obtained by analyzing either aerosol particles or cloud water. When analyzing cloud water, the mass concentration of ${\text{SO}}_{4}^{2-}$ dissolved in droplets (mg L^−1^) is converted to the air-equivalent mass concentration (μg m^−3^) by multiplying by the liquid water content, LWC (g m^−3^), in a cloud. The data used to create $N_{\text{d}}-{\text{SO}}_{4}^{2-}$ empirical parameterizations are typically derived from field campaigns, which differ in the region of analysis, sampling platforms (aircraft or ground-based), measurement approach (e.g., in particle form or dissolved in cloud water), and number of species analyzed. While the literature evaluating relationships between cloud water composition and *N*_d_ is limited and largely from aircraft studies from more than a decade ago, there is a growing number of data sets characterizing *N*_d_ and cloud water composition that are of interest to continue this line of research. Examples include the recently completed Cloud, Aerosol, and Monsoon Processes Philippines Experiment (CAMP^2^Ex), the North Atlantic Aerosols and Marine Ecosystems Study (NAAMES) (Behrenfeld et al., 2019), and the current multiyear Aerosol Cloud meTeorology Interactions oVer the western ATlantic Experiment (ACTIVATE) (Sorooshian et al., 2020). A summary of relevant past field work follows.

Leaitch et al. (1986) sampled continental stratiform and cumuliform clouds over Ontario, Canada, and showed a roughly linear relationship between *N*_d_ and ${\text{SO}}_{4}^{2-}$ at low ${\text{SO}}_{4}^{2-}$ concentrations (below 5 μg m^−3^), and they showed that the relationship leveled out at higher concentrations (Novakov et al., 1994). Leaitch et al. (1992) suggested that the low *R*^2^ values for the linear regression between *N*_d_ and ${\text{SO}}_{4}^{2-}$ for both continental stratiform and cumuliform clouds (0.30 and 0.49, respectively) stemmed from factors such as (i) other chemical species besides ${\text{SO}}_{4}^{2-}$ and variability in both (ii) updraft wind speed and (iii) temperature. Pueschel et al. (1986) sampled clouds originating from marine and continental air masses at a ground-based observatory at White-face Mountain, New York. They found that emissions contributed strongly to ${\text{SO}}_{4}^{2-}$ and that a significant portion of ${\text{SO}}_{4}^{2-}$-containing particles acted as CCN and thus likely impacted *N*_d_. Novakov et al. (1994) sampled marine cumulus and stratocumulus clouds by El Yunque peak in Puerto Rico. Although they showed that *N*_ccn_ and ${\text{SO}}_{4}^{2-}$ were highly correlated in both cumulus and stratocumulus clouds, they also found that *N*_d_ and ${\text{SO}}_{4}^{2-}$ were weakly correlated for stratocumulus clouds and not correlated for cumulus clouds. They attributed this difference to the effect of entrainment and mixing on cloud microphysics. Leaitch et al. (1996) sampled marine stratus clouds over the Gulf of Maine and the Bay of Fundy during the North Atlantic Regional Experiment (NARE) and showed that ${\text{SO}}_{4}^{2-}$ was better correlated with *N*_d_ than nitrate (${\text{NO}}_{3}^{-}$) (with *R*^2^ values of 0.30 and 0.12, respectively). The *R*^2^ between *N*_d_ and ${\text{SO}}_{4}^{2-}$ increased when the data were stratified into bins of low and high turbulence, which was quantified as the standard deviation of vertical wind speed. They found that in situations with lower supersaturations, *N*_d_ was more influenced by turbulence than by either ${\text{SO}}_{4}^{2-}$ or *N*_a_. Menon and Saxena (1998) and Saxena and Menon (1999) sampled orographic clouds at a ground-based station at Mt. Mitchell, North Carolina. They found that ${\text{SO}}_{4}^{2-}$ was the main contributor to cloud water acidity and a reliable tracer for anthropogenic pollution. Log-log regressions of ${\text{SO}}_{4}^{2-}$−*N*_d_ were binned according to the level of ${\text{SO}}_{4}^{2-}$, with not much difference observed between the different levels of pollution. Borys et al. (1998) and Lowenthal and Borys (2000) sampled warm marine stratiform clouds on the island of Tenerife in the Canary Islands. They found that *N*_d_ was influenced by ${\text{NSS-SO}}_{4}^{2-}$, ${\text{NO}}_{3}^{-}$, pollution-derived trace elements, and elemental carbon (EC), signifying that species other than ${\text{SO}}_{4}^{2-}$ influenced *N*_d_. Despite the sampling site proximity to African deserts, the mass concentration of crustal elements contained in dust was found to have little correlation with *N*_d_. Also, the sea salt tracer sodium (Na^+^) was found to have little correlation with *N*_d_. Several studies (e.g., Boucher and Lohmann, 1995; Lowenthal et al., 2004; Menon et al., 2002; Van Dingenen et al., 1995) have combined field data, such as those mentioned above, in addition to other data sets, with the intention of producing a robust empirical prediction of *N*_d_. Menon et al. (2002) provided a log-log multispecies prediction of *N*_d_ using ${\text{SO}}_{4}^{2-}$, organic matter, and sea salt. Organic carbon has been shown to increase *N*_d_, as it affects the surface tension of cloud droplets (e.g., Facchini et al., 1999; Nenes et al., 2002). Additionally, nitric acid (HNO_3_) has been linked with increased CCN activity and *N*_d_ based on modeling studies (Hegg, 2000; Kulmala et al., 1993; Xue and Feingold, 2004).

McCoy et al. (2017) used *N*_d_ data from the Moderate Resolution Imaging Spectroradiometer (MODIS) satellite instead of in situ measurements. Second, aerosol mass concentration data were obtained from the Modern-Era Retrospective analysis for Research and Applications version 2 (MERRA-2; Gelaro et al., 2017) reanalysis product and various aerosol transport models instead of in situ measurements. Third, the study region was more global in nature (albeit focusing on marine stratocumulus clouds) instead of a specific region. Fourth, since reanalysis data were used, a multispecies, multi-variable linear regression was performed:

(2) $$\text{log}\left(N_{\text{d}}\right)=a_{0}+a_{1}\text{ log}\left({\text{SO}}_{4}^{2-}\right)+a_{2}\text{ log}(\text{SS})\;+a_{3}\text{ log}(\text{BC})+a_{4}\text{ log}(\text{OC})+a_{5}\text{ log}(\text{DU}),$$

where SS is sea salt, BC is black carbon, OC is organic carbon, and DU is dust. McCoy et al. (2017) found that ${\text{SO}}_{4}^{2-}$ was predominantly correlated with *N*_d_, with sea salt, black carbon, organic carbon, and dust accounting for smaller contributions. A caveat to consider when comparing the findings of McCoy et al. (2018) to other aircraft studies is that McCoy et al. (2018) used mass concentrations retrieved exclusively at the 910 hPa model level (~ 915 m) and only considered mass concentrations pertaining to submicron SS/DU and hydrophilic BC/OC.

The field studies cited above still leave a series of unanswered questions that the current study aims to address there: (i) how is the ${\text{SO}}_{4}^{2-}$−*N*_d_ relationship affected by vertical wind speed (Leaitch et al., 1992), turbulence (Leaitch et al., 1996), and entrainment (Novakov et al., 1994); (ii) why do species such as sea salt and dust play such a minor role in influencing *N*_d_, even when located over the ocean and near a desert (Borys et al., 1998; McCoy et al., 2017, 2018); (iii) what is the relationship between organic matter and *N*_d_ (McCoy et al., 2018; Nenes et al., 2002); and (iv) can the ${\text{SO}}_{4}^{2-}$‒*N*_d_ correlation be improved by considering other chemical species (e.g., Hegg et al., 1993; Leaitch et al., 1992; Novakov and Penner, 1993). The present study will examine these questions using a data set comprised of in situ aircraft measurements collected off the California coast during four field campaigns. In addition to meteorological and aerosol and cloud microphysical measurements, a total of 385 cloud water samples were collected and analyzed for 80 chemical species (ions and elements). Even though measurements were collected in only one localized region, it is expected that the variety of conditions encountered over four summers, together with the large number of chemical species analyzed, will help address the questions noted above. The results of this work have implications for simulations and retrievals of *N*_d_, in addition to studies examining relationships between atmospheric chemistry and cloud microphysics.

## 2 Methodology

### 2.1 Aircraft campaigns and study region

**Figure 1.**
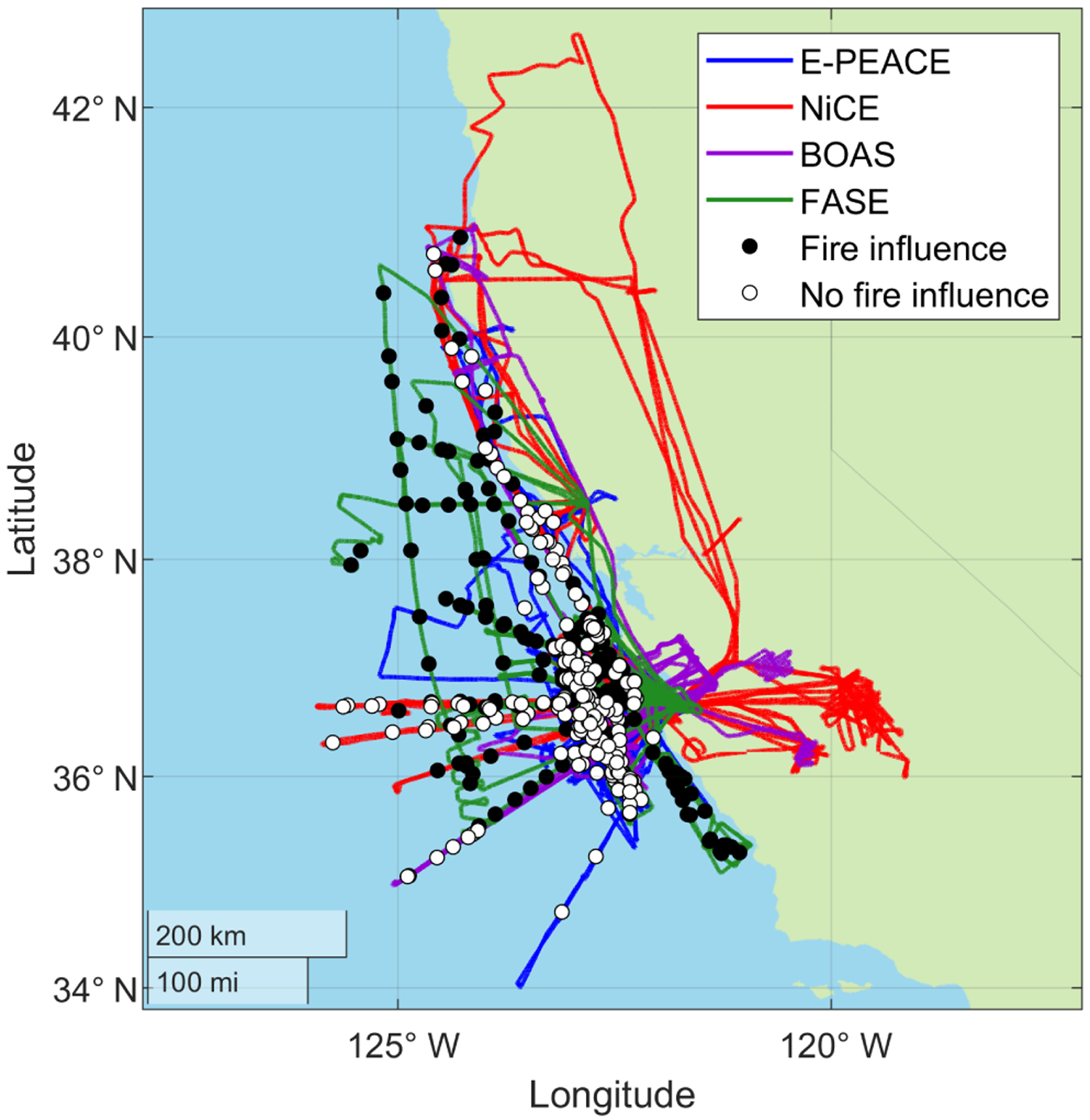
Flight paths for each of the four campaigns used in this study. Markers indicate the average location at which the cloud water samples were collected. Smoke- and non-smoke-influenced samples are indicated with filled and open markers, respectively.

This work reports results relevant to warm marine stratocumulus clouds off the California coast based on field measurements from four field campaigns between 2011 and 2016, each during the months of July and August. The persistent summertime stratocumulus cloud deck located off the California coast offers the ideal natural laboratory to study aerosol-cloud-precipitation-meteorology interactions (Russell et al., 2013; Sorooshian et al., 2018). For all field campaigns, the Center for Interdisciplinary Remotely-Piloted Aircraft Studies (CIRPAS) Twin Otter was deployed out of Marina, California, with an almost identical instrumentation payload. The four campaigns addressed in this study are the Eastern Pacific Emitted Aerosol Cloud Experiment (E-PEACE) (Russell et al., 2013; Wonaschütz et al., 2013), the Nucleation in California Experiment (NiCE) (Crosbie et al., 2016; Maudlin et al., 2015), the Biological and Oceanic Atmospheric Study (BOAS) (Wang et al., 2016), and the Fog and Stratocumulus Evolution (FASE) experiment (Dadashazar et al., 2017; MacDonald et al., 2018). Research flight information and tracks are shown in Table 1 and Fig. 1, respectively.

Previous studies have used back-trajectory analysis to show that air in the MBL in the study region is predominantly influenced by air mass transport from the north and northwest (Schlosser et al., 2020; Wang et al., 2016; Wonaschütz et al., 2013). Thus, the cloud water in this study was influenced by a variety of local and long-range sources such as ship exhaust (Chen et al., 2012; Coggon et al., 2012), biomass burning (Prabhakar et al., 2014; Mardi et al., 2018), ocean emissions (Dadashazar et al., 2017; MacDonald et al., 2018), continental pollution (Ma et al., 2019; Wang et al., 2016), and dust (Mardi et al., 2019; Wang et al., 2014).

### 2.2 Aircraft instrumentation

Aircraft instrumentation used in each campaign is described in detail in Sorooshian et al. (2018). The relevant instrumentation used in the present study is as follows: aerosol size distribution was measured using a passive cavity aerosol spectrometer probe (PCASP; particle diameter (*D*_p_) of ~ 0.1–2.6 μm; Strapp et al., 1992); cloud droplet size distribution was measured using a forward scattering spectrometer probe (FSSP; *D*_p_ of ~ 2–45 μm; Gerber et al., 1999) and a cloud and aerosol spectrometer - forward scattering (CASF; *D*_p_ of ~ 1–61 μm; Baumgardner et al., 2001); rain drop size distribution was measured using a cloud imaging probe (CIP; *D*_p_ of ~ 25–1600 μm; Baumgardner et al., 2001); cloud liquid water content (LWC) was measured using a particulate volume monitor (PVM-100A; *D*_p_ of ~ 3–50 μm; Gerber, 1994); and three-dimensional wind speeds were calculated by combining the pressure measurements from a five-hole Radome gust probe plumbed into the aircraft nose together with the aircraft velocity and altitude measurements provided by the aircraft’s Global Positioning System and inertial navigation system (GPS/INS).

Since LWC played a critical role in converting aqueous concentration to air-equivalent concentration, the size range used to calculate *N*_d_ was bracketed to resemble the size range of the PVM-100A. Therefore, *N*_d_ was defined in this study to be equivalent to the integration of the cloud droplet size distribution between *D*_p_ of ~ 3–50 μm and was calculated using CASF (for E-PEACE) and FSSP (NiCE, BOAS, and FASE). For the NiCE campaign, LWC measurements from the PVM-100A instrument were unreliable; therefore, the LWC for NiCE was calculated instead using FSSP data between *D*_p_ of ~ 3–50 μm.

### 2.3 Cloud water collection and chemical analysis

A total of 385 cloud water samples were collected throughout the four campaigns using a modified Mohnen slotted-rod collector, reported to collect droplets with *D*_p_ of ~ 5–35 μm (Hegg and Hobbs, 1986). The cloud water was collected in polyethylene bottles and stored at ~ 5° C for subsequent offline chemical analysis. The spatially averaged location of each cloud water sample is shown in Fig. 1. Cloud water samples were chemically analyzed post-flight for ions using ion chromatography (IC; Dionex ICS-2100) and for elements using inductively coupled plasma mass spectrometry (ICP-MS; Agilent 7700 Series) for E-PEACE, BOAS, and NiCE or triple quadrupole inductively coupled plasma mass spectrometry (ICP-QQQ; Agilent 8800 Series) for FASE. The limit of detection (LOD) for each ion and element measured is shown in Table S1 in the Supplement. The concentration of non-sea-salt (NSS) species was calculated using the relative abundance of a NSS species to Na^+^ in natural sea salt (Seinfeld and Pandis, 2016). Cloud water sample acidity was quantified by measuring pH (the aqueous concentration of hydrogen ions, H^+^) using a Thermo Scientific Orion 9110DJWP Combination Semi-Micro pH electrode for E-PEACE, NiCE, and BOAS and a Thermo Scientific Orion 8103BNUWP Ross Ultra Semi-Micro pH probe for FASE. Aqueous concentrations (i.e., mass concentrations in the droplets, mg L^−1^) were converted to air-equivalent concentrations (i.e., mass concentrations in the air, μg ${\text{m}}_{\text{air}}^{-3}$) by multiplying aqueous concentrations by the LWC and dividing by the mass density of water. This study uses air-equivalent concentrations for all species with the exception of H^+^ (pH) that uses aqueous concentration.

A total of 80 species (29 measured ionic species, 46 measured elemental species, measured pH, and 4 NSS calculated species; Table 2) were considered in this study as an initial pool of candidate species that could potentially be used to predict *N*_d_. To facilitate the statistical analysis in this study, the amount of chemical species were filtered from 80 to only 9. The steps used in this filtering process are summarized in the next section.

### 2.4 Filtering of chemical species

A focus in this study is to identify appropriate chemical species to use as predictors in a linear regression model (addressed in Sect. 2.5). Good statistical practice (e.g., Freund et al., 2010) recommends that two conditions must be met to produce a meaningful multivariable regression: (1) the independent/predictor variables must not be redundant, i.e., they must not be highly correlated among themselves (the property of high correlation is called collinearity), and (2) each independent/predictor variable must have some correlation with the dependent/response variable. There is no universal rule to define what is “highly” correlated; rather, it depends on the nature of the data and the user’s judgment.

**Figure 2.**
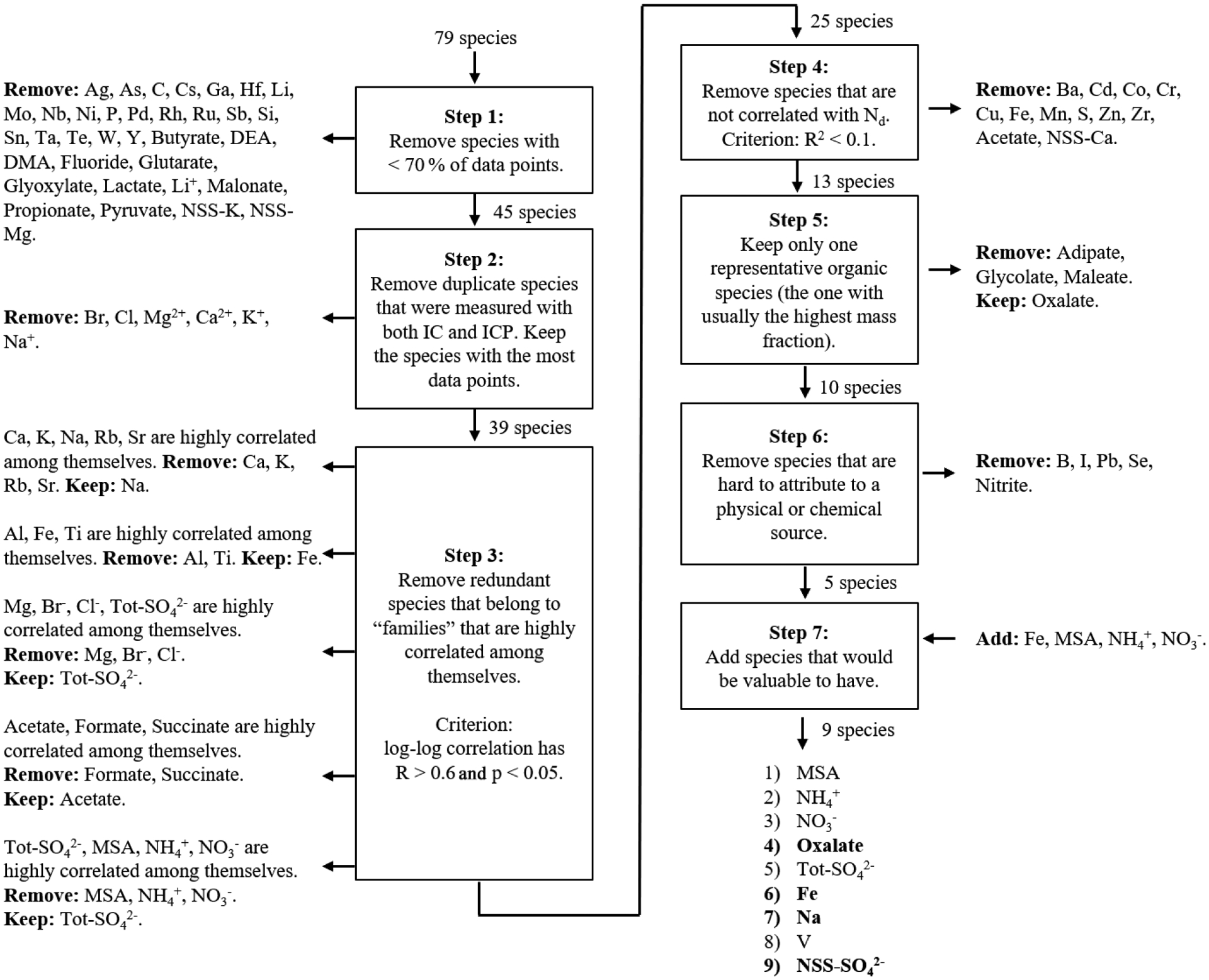
Algorithm used to filter the number of species from 80 to 9. The four species in bold font are the ones used in Sect. 3.3. ICP represents ICP-MS + ICP-QQQ.

As using all 80 species is impractical in terms of providing results that could be tested and/or used by others, a filtering method was used to reduce the number of species. The filtering method consisted of seven steps (Fig. 2), the objective of which was to trim the total number of species by an order of magnitude, leaving just a few that exhibited the following conditions: (1) the highest data quality and quantity, (2) the least redundancy among themselves, (3) the highest correlation with *N*_d_, and (4) the most physical meaning. The decision to remove a species becomes less objective and quantifiable towards the last steps in Fig. 2. Each step is described below.

Step 1 removed species with less than 70 % of data points. A species could have a low amount of points because it was not analyzed in a field campaign or because the data quality from the IC or ICP (ICP-MS or ICP-QQQ) was inadequate. Step 2 removed duplicate species that were measured by both IC and ICP. Step 3 addressed condition (2) by removing species that were collinear (i.e., correlated among themselves). The criterion for a “high” correlation was to have a correlation coefficient (*R*) > 0.6 and a *p* value < 0.05. For example, if a fixed number of five species were all highly correlated between each other, then only one of the five species was kept and the rest were removed. This procedure is to consolidate “families” of three or more highly correlated species to a single species and does not apply to pairs of highly consolidated species. Step 4 addressed condition (3) by removing species that were not correlated to *N*_d_. The criterion for a “low” correlation was to have a coefficient of determination (*R*^2^) < 0.1. Notice that step 3 uses *R*, whereas step 4 uses *R*^2^; this is because collinearity is determined not only by the value of *R* but also the sign of *R*. Step 5 removes all but one organic species, oxalate (Ox), since this species generally had the highest mass concentration of all the organic species and was considered to be representative of all other organic species. Step 6 removed species that could not easily be attributed to a physical process or chemical source. Step 7 added back into the analysis four species that had been removed. This was done for the sake of having species that are known to have relevant sources in the study region. Even though pH plays an important role in the partitioning of gases into particles and droplets, in addition to influencing aqueous reactions in droplets (e.g., Pye et al., 2020), pH was filtered out in step 4 for being a poor predictor of *N*_d_.

The nine species that survived the filtering scheme in Fig. 2 are methanesulfonic acid (MSA), ammonium (${\text{NH}}_{4}^{+}$), ${\text{NO}}_{3}^{-}$, Ox, ${\text{Tot-SO}}_{4}^{2-}$, ${\text{NSS-SO}}_{4}^{2-}$, Fe, Na, and vanadium (V). These species have known sources as follows. MSA: ocean biogenic (Sorooshian et al., 2009); ${\text{NH}}_{4}^{+}$: agriculture (Bauer et al., 2016), marine emissions (Bouwman et al., 1997), and wildfires (Reid et al., 1998); ${\text{NO}}_{3}^{-}$ and Ox: fire (Prabhakar et al., 2014; Maudlin et al., 2015); ${\text{Tot-SO}}_{4}^{2-}$: sea salt (Seinfeld and Pandis, 2016), ocean biogenic (Charlson et al., 1987), and shipping (Coggon et al., 2012), with ${\text{NSS-SO}}_{4}^{2-}$ missing the sea salt contribution; Fe: dust (Jickells et al., 2005) and fire (Maudlin et al., 2015); Na: sea salt (Seinfeld and Pandis, 2016); and V: shipping (Wang et al., 2014). Note that we retained both ${\text{Tot-SO}}_{4}^{2-}$ and ${\text{NSS-SO}}_{4}^{2-}$; this is to evaluate which correlates more with *N*_d_, as some studies have used ${\text{Tot-SO}}_{4}^{2-}$ (e.g., Leaitch et al., 1992; Saxena and Menon, 1999), whereas others have used ${\text{NSS-SO}}_{4}^{2-}$ (Novakov et al., 1994; Boucher and Lohmann, 1995). Section 3.1 and 3.2 will discuss these nine species, and the rest of Sect. 3 will focus on only four species to be explained later. These species were analyzed by a multivariable regression model, which is described in the next section.

### 2.5 Mathematical model

This study examines the relationship between cloud water mass concentration and *N*_d_ with a multivariable linear model similar to that of McCoy et al. (2017, 2018):

(3) $$\text{log}\left(N_{\text{d}}\right)=a_{0}+a_{1}\text{ log}\left(M_{1}\right)+a_{2}\text{ log}\left(M_{2}\right)\;+\ldots +a_{n}\text{ log}\left(M_{n}\right),$$

where *M_i_* is the air-equivalent mass concentration of species *i* (μg m^−3^), *a_i_* represent fitting parameters, and *n* is the number of species being considered. *N*_d_ is the dependent (or response) variable and *M*_1_, *M*_2_, …, *M*_*n*_ are the independent (or predictor) variables. The logarithmic forms of *N*_d_ and *M_i_* were correlated to account for a numerically large range of several orders of magnitude and because a log-log model is commonly used to correlate chemical composition to *N*_d_ (e.g., Boucher and Lohmann, 1995; Menon et al., 2002; McCoy et al., 2017).

The MATLAB software package was used to obtain multivariable linear regressions of the form of Eq. (3) using the method of ordinary least squares. The performance of a regression was quantified using the coefficient of determination (*R*^2^). However, when comparing the performance of correlations between regressions using a different number of predictor variables, it is necessary to use the adjusted coefficient of determination ($R_{\text{adj}}^{2}$), which is subscripted to distinguish it from the ordinary *R*^2^, and is adjusted by using the number of predictors (*P*) and the number of data points (*N*) via the formula $R_{\text{adj}}^{2}=1-\left(1-R^{2}\right)(N-1)/(N-P-1)$ (Kahane, 2008). For a large number of data points, $R_{\text{adj}}^{2}\approx R^{2}$; however, for the sake of rigor and consistency, $R_{\text{adj}}^{2}$ is used instead of the ordinary *R*^2^, except when reporting values from the literature. The statistical significance of correlations was quantified using the *p* value obtained by doing a two-tailed Student’s *t* test. Both $R_{\text{adj}}^{2}$ and *p* values were given by the MATLAB software after regression. *p* values were obtained for both the overall regression and each individual coefficient in the regression, e.g., if a regression has three predictors, there are a total of five *p* values: one for the overall regression, three for the slope of each individual predicting variable, and one for the intercept. In this study, a regression was considered to be statistically significant if all the *p* values were < 0.05.

The correct functioning of the method of ordinary least squares requires that the set of *n* predicting variables in Eq. (3) not be collinear. Multicollinearity is defined by a set of three or more predicting variables being collinear. Using a set of multicollinear predictors can produce unreliable estimates in both magnitude and sign of the coefficients (*a_i_*) (Kahane, 2008). There is no universal marker for multicollinearity. Furthermore, multicollinearity can only be addressed when analyzing all predictors together. For example, for a given set of three predictors (*P*_1_, *P*_2_, and *P*_3_), even though the pairs *P*_1_ – *P*_2_, *P*_1_ – *P*_3_, and *P*_2_ – *P*_3_ are not collinear, there is no guarantee that the *P*_1_ – *P*_2_ – *P*_3_ set is not multicollinear. When considering a complex system such as the chemical composition of cloud water, it is reasonable to assume that as more species are used to predict *N*_d_, the higher the probability that the set of species is multicollinear. We did not test for multicollinearity in this study; the consequences of not doing so are explored in Sect. 3.2.

### 2.6 Calculation of turbulence

Similar to Leaitch et al. (1992) and Feingold et al. (1999), this study analyzes the effect of turbulence on the ability to predict *N*_d_. Turbulence was considered to be represented by the standard deviation of the vertical wind speed (*w*) and is represented as *σ_w_*. Also similar to Leaitch et al. (1992), this study classified conditions into turbulent and smooth regimes by considering the upper and lower 33rd percentile of *σ_w_*, respectively. Although the rigorous approach to calculate *σ*_*w*_ uses *w* from below the cloud (Twomey, 1959), this study used vertical wind speed data collected throughout the sampling time (i.e., mostly inside the cloud but also outside the cloud). This was mainly because not all cloud water samples had an accompanying measurement of *w* below the cloud. To justify using *σ*_*w*_ from the sampling time instead of below-cloud *σ*_*w*_, consider Fig. S1 in the Supplement, which shows a representative time series of altitude, *w*, and *σ*_*w*_ for a cloud water sample that was collected minutes before a below-cloud leg, which collected measurements of *w*. It can be seen that the plots of *w* and aw are similar and that an average *σ*_*w*_ calculated either way is still in the bottom 33rd percentile. Therefore, for the purposes of this study, we consider in-cloud turbulence to reasonably approximate below-cloud turbulence.

### 2.7 Determination of smoke influence

One of the objectives of this study is to analyze the extent to which the presence of smoke from wildfires affects the correlation between *N*_d_ and cloud water chemical composition. Thus, it was important to identify cloud water samples that were influenced by smoke. Only the NiCE and FASE campaigns were affected by wildfires. Mardi et al. (2018) identified vertical soundings in the NiCE and FASE campaigns that were influenced by smoke by establishing smoke influence to have a total aerosol number concentration (*N*_a_) ≥ 1000 cm^−3^, as measured by the PCASP, in addition to visual and olfactory detection of smoke by flight scientists. In this study, a cloud water sample was considered to be influenced by smoke if it was collected during a research flight (RF) that contains a vertical sounding identified by Mardi et al. (2018) to be influenced by smoke, even if the cloud water sample was not necessarily collected near the sounding labeled as smoke influenced; this is a valid assumption based on the work of Mardi et al. (2019). The RFs considered to be smoke influenced in this study were NiCE RFs 16–23 and FASERFs 3–11 and 13–15.

## 3 Results and discussion

With the refined list of nine physically meaningful species from Sect. 2.4, we now proceed to address the following questions: (1) what single species best predicts *N*_d_; (2) how many species are sufficient to predict *N*_d_; (3) what is an effective combination of species to predict *N*_d_; and (4) how do several factors (i.e., turbulence, smoke-influence, and location along cloud depth) affect the ability to reliably predict *N*_d_. These questions are addressed in order in Sect. 3.1–3.4.

### 3.1 Single-variable prediction of *N*_d_

**Figure 3.**
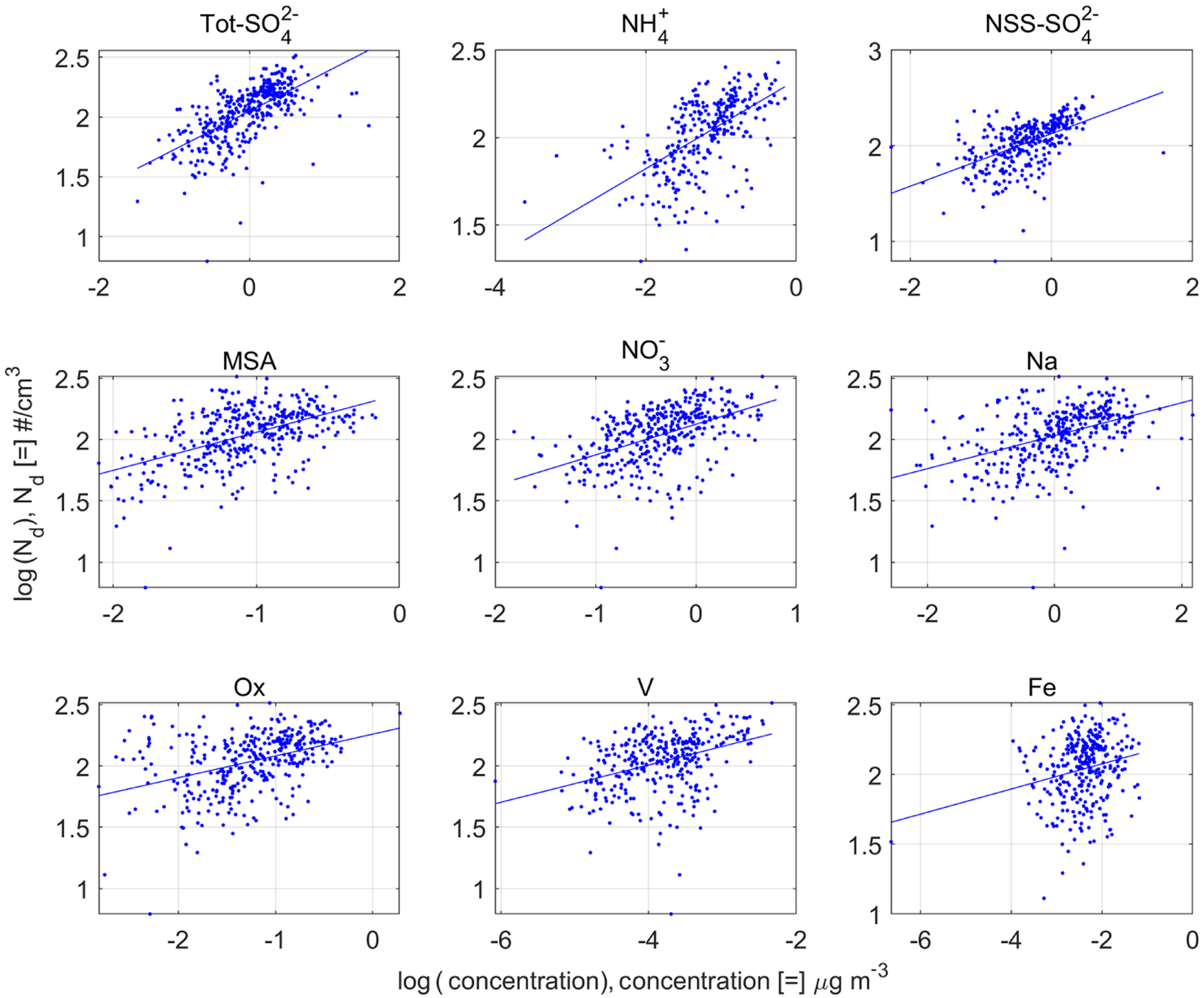
Scatter plot for the nine filtered species from Fig. 2. The lines are linear regression models of the form log(*N*_d_) = *a*_0_ + *a*_1_ log(*M_i_*), where *M*_*i*_ is the mass concentration of species *i*.

In this section, we analyze which of the nine species filtered out in Sect. 2.4 best predicts *N*_d_ by itself without binning by external factors. These single-predictor regressions with no binning are important, as they provide a baseline for subsequent sections in which multi-predictor regressions and binning are used. Table 3 and Fig. 3 display the ability of each of the nine species to predict *N*_d_. To have consistency with subsequent sections, $R_{\text{adj}}^{2}$ is used instead of the ordinary *R*^2^. The regression and the individual coefficients were all statistically significant.

Some previous studies predicted *N*_d_ using ${\text{Tot-SO}}_{4}^{2-}$ (e.g., Leaitch et al., 1992; Saxena and Menon, 1999), whereas other studies used ${\text{NSS-SO}}_{4}^{2-}$ (e.g., Novakov et al., 1994; Lowenthal et al., 2004). We find that ${\text{Tot-SO}}_{4}^{2-}$ is the best predictor and that it is better correlated to *N*_d_ ($R_{\text{adj}}^{2}=0.40$) than ${\text{NSS-SO}}_{4}^{2-}$ ($R_{\text{adj}}^{2}=0.29$). This is likely because ${\text{Tot-SO}}_{4}^{2-}$ encompasses both sea salt particles and non-sea-salt particles and thus gives a better approximation to the total number concentration of CCN. In addition, ${\text{Tot-SO}}_{4}^{2-}$ also had the largest slope (*a*_1_ = 0.32), suggesting that *N*_d_ is more sensitive to changes in ${\text{Tot-SO}}_{4}^{2-}$ than other chemical species. Although HNO_3_ has been observed to increase *N*_d_ (e.g., Xue and Feingold, 2004), ${\text{NO}}_{3}^{-}$ was found to be only moderately correlated with *N*_d_ ($R_{\text{adj}}^{2}=0.24$). The species with the lowest correlation was Fe ($R_{\text{adj}}^{2}=0.05$). This low correlation with *N*_d_ was also presented by other crustal metals like Al ($R_{\text{adj}}^{2}=0.01$) and Ti ($R_{\text{adj}}^{2}\sim 0$) (not shown in Table 3). The low influence of crustal metals on *N*_d_ is consistent with the findings of Lowenthal and Borys (2000). Some physical meaning can be extracted from the intercept of the regression (*a*_0_). If *N*_d_ is insensitive to the mass concentration of a species, then the slope (*a*_1_) should be zero, and *N*_d_ would be constant with a value of *N*_d_ = 10^*a*0^. These intercepts yield a range of *N*_d_ of 108–412cm^−3^. These values are not unrealistic in clouds in this study region (e.g., Chen et al., 2012; Lu et al., 2009; Wang et al., 2016).

To contrast with results of this work, Table 4 shows the regression parameters from other studies when correlating *N*_d_ and ${\text{SO}}_{4}^{2-}$. For the sake of completeness, Table 4 shows regressions that analyzed non-marine stratocumulus clouds, but in this comparison, we focus only on those regressions that analyzed stratocumulus clouds. Our results (i.e., *a*_*i*_ coefficients and *R*^2^) for ${\text{Tot-SO}}_{4}^{2-}$ reasonably match the results of Leaitch et al. (1992), suggestive of commonality between two coastal regions with differing meteorological conditions (i.e., northeast Pacific vs northwest Atlantic) (Sorooshian et al., 2019). Our results for ${\text{NSS-SO}}_{4}^{2-}$ also reasonably match those of McCoy et al. (2017), which is noteworthy as McCoy et al. (2017) used satellite retrievals and model aerosol concentrations for several stratocumulus decks around the world, whereas our analysis used in situ data from a relatively small region. However, our ${\text{NSS-SO}}_{4}^{2-}$ results differ significantly from those of Novakov et al. (1994), which is understandable since the regression presented by Novakov et al. (1994) has a *p* value > 0.05. Our data set does not achieve the degree of correlation achieved by Lowenthal et al. (2004), who report the highest correlation for marine clouds (*R*^2^ = 0.82). The studies that analyzed stratocumulus clouds all report intercept values (*a*_0_) of ~ 2.0, which is consistent with our data.

### 3.2 Multi-variable prediction of *N*_d_

When previous studies correlated *N*_d_ (or *N*_CCN_) and the air-equivalent concentration of chemical species and obtained a poor correlation, it was suggested that taking more chemical species into consideration would improve the correlation (e.g., Leaitch et al., 1992; Novakov et al., 1994). In this section we address the following issue: “How many chemical species are necessary to adequately predict *N*_d_”. To answer this question, we use the nine filtered species from Sect. 2.4. Regressions of the form of Eq. (3) are performed for every combination of species. The number of predictors in the regressions are varied from one up to eight. The number of combinations (*C*) that can be made with *P* predictors selected from *S* species is *C* = *S*!/(*P*!(*S* – P)!). Combinations that include ${\text{Tot-SO}}_{4}^{2-}$ and ${\text{NSS-SO}}_{4}^{2-}$ together are not considered, thus leaving a total of 383 regressions.

**Figure 4.**
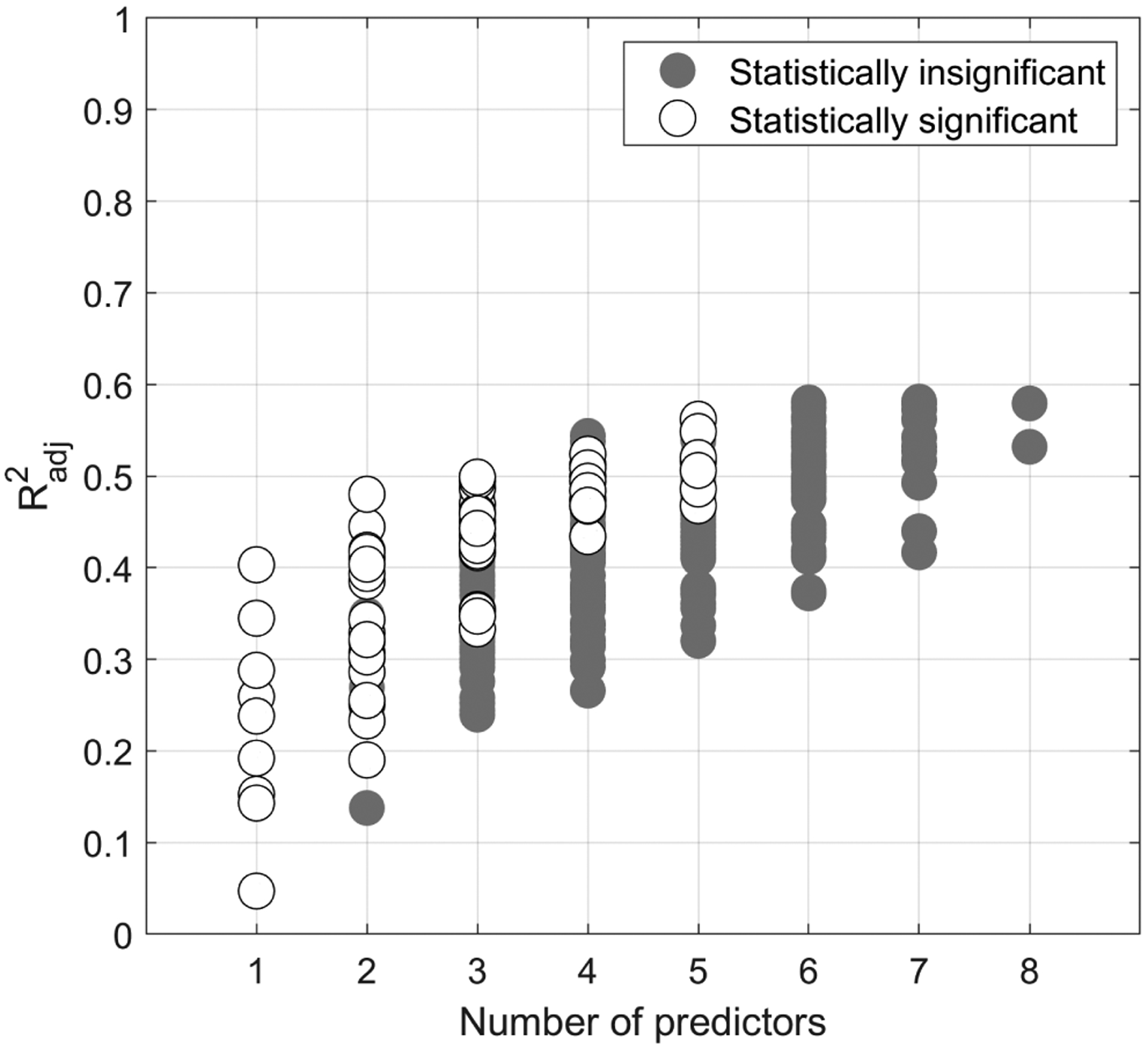
Plot showing which of the 383 regressions are statistically significant. This plot ignores the regressions that use both ${\text{NSS-SO}}_{4}^{2-}$ and ${\text{Tot-SO}}_{4}^{2-}$ simultaneously.

Of the total 383 regression, only 67 were considered statistically significant. Figure 4 shows the $R_{\text{adj}}^{2}$ as a function of the number of predictors for both statistically significant and insignificant regressions; the percentage of regressions that were statistically significant is shown in Table S2. These results show that adding more predictors does not necessarily improve the correlation, as all correlations that use six or more predictors are statistically insignificant. This behavior is perhaps because the new species being added are redundant with respect to the species that are already in the model (i.e., the new species is mathematically collinear with the old species). It is also interesting to note how $R_{\text{adj}}^{2}$ increases asymptotically to ~ 0.6; this further makes the point that additional species do not necessarily improve predictability of *N*_d_. The same asymptotic behavior is also exhibited with *R*^2^, as *R*^2^ and $R_{\text{adj}}^{2}$ for these regressions differ by only ~ 2 %.

We examined the best regressions produced by a given number of predictors to explore the factors that contribute to a respectable multivariable regression. Table 5 shows the three statistically significant regressions that had the highest $R_{\text{adj}}^{2}$ for a given number of predictors (one to five). The predictors are ordered horizontally according to the value of their coefficient in order to show qualitatively which species is more dominant in a regression. Eight of the nine chemical species considered appear at least once in a regression, with the most common species being ${\text{NH}}_{4}^{+}$, a form of ${\text{SO}}_{4}^{2-}$ (total or non-sea-salt), Na, Ox, and MSA. Sulfate (total or non-sea-salt) appears in 12 of the 15 regressions, and in eight regressions it has the largest coefficient; this speaks to the importance of ${\text{SO}}_{4}^{2-}$ in predicting *N*_d_. However, the appearance of Na and Ox and their non-negligible slope also highlights the importance of considering them as well in a correlation; this is clearly observed in the increase of $R_{\text{adj}}^{2}$ when Na and Ox are added to a regression that contains only ${\text{NSS-SO}}_{4}^{2-}$ (Table 6). We believe that the ingredients that yield the higher $R_{\text{adj}}^{2}$ in Table 5 are (1) a form of $R_{\text{adj}}^{2}$ (such ${\text{Tot-SO}}_{4}^{2-}$ or ${\text{NSS-SO}}_{4}^{2-}$), (2) a sea emissions tracer (such as Na), and (3) an organic tracer (such as Ox). ${\text{NH}}_{4}^{+}$ was present in all the regressions; however, given that it comes from diverse sources such as agriculture (ApSimon et al., 1987; Bauer et al., 2016), marine emissions (Bouwman et al., 1997; Paulot et al., 2015), and wildfires (Maudlin et al., 2015; Reid et al., 1998), it is difficult to assess if it contributes to the CCN budget or simply accompanies all types of CCN. In other words, we suspect that ${\text{NH}}_{4}^{+}$ appears in all correlations because it generally accompanies the three ingredients we propose make a good correlation: a form of ${\text{SO}}_{4}^{2-}$, a marine emissions tracer, and an organic tracer.

It is of interest to note that combining a sea salt tracer (such as Na) with ${\text{NSS-SO}}_{4}^{2-}$ in a two-predictor model has about the same performance ($R_{\text{adj}}^{2}=0.41$; Table 6) as a one-predictor model using ${\text{Tot-SO}}_{4}^{2-}$ ($R_{\text{adj}}^{2}=0.40$; Table 3). We believe this is because ${\text{Tot-SO}}_{4}^{2-}$ encompasses the sea salt and the non-sea-salt contribution to CCN about the same as the artificial mathematical separation of the two. Also of interest is that when only looking at the statistically significant regressions, only 17 regressions have species with negative coefficients (i.e., negative slopes). The species with negative coefficients are ${\text{NO}}_{3}^{-}$, Fe, and V (not shown); more specifically, ${\text{NO}}_{3}^{-}$, Fe, and V have negative coefficients when they are accompanied by ${\text{NH}}_{4}^{+}$ in the same regression. The physical reason as to why these species have negative coefficients when mixed with ${\text{NH}}_{4}^{+}$ is not clear; perhaps the reason is due to the mathematics of the regression and not physically rooted, as multicollinearity can lead to unexpected magnitudes and signs for predictor coefficients (Kahane, 2008). In addition, multicollinearity will become more likely as more predictors as considered. Therefore, it is not surprising that unexpected negative coefficients only appear when considering many (five) predictors. Lastly, a correlation matrix among the nine predicting species (Fig. S2) shows a strong correlation for some pairs of species (${\text{NH}}_{4}^{+}-{\text{NO}}_{3}^{-}:R_{\text{adj}}^{2}=0.48;{\text{NO}}_{3}^{-}-\text{V}:R_{\text{adj}}^{2}=0.49$) and moderate correlation for other pairs (${\text{NH}}_{4}^{+}-\text{V}:R_{\text{adj}}^{2}=0.27;{\text{NO}}_{3}^{-}-\text{Fe}:R_{\text{adj}}^{2}=0.22$), thus strengthening the argument that the negative coefficients are due to mathematical multicollinearity and not a physical or chemical reason.

When considering a multispecies model to predict *N*_d_, it is worthwhile to examine the coefficient of sea salt. Even though it is well established that more CCN leads to more droplets, the effect of giant CCN (GCCN), such as sea salt, is not as clear. Cloud microphysics studies suggest two mechanisms by which more sea salt leads to less *N*_d_. (1) The large size and highly hygroscopic nature of sea salt causes these particles to activate into droplets before other smaller particles. This reduces the amount of available water vapor and creates unfavorable conditions for smaller particles to nucleate into droplets (e.g., Andreae and Rosenfeld, 2008). (2) GCCN nucleate into larger droplets as compared to CCN, which in turn are more likely to collide and coalesce with surrounding droplets. This combination of droplets creates larger but fewer droplets and ultimately leads to the formation of rain drops and precipitation (e.g., Feingold et al., 1999; Jung et al., 2015). Therefore, it is expected that the negative correlation between GCCN and *N*_d_ should translate into a negative coefficient for Na (the sea salt tracer) in a multi-predictor regression equation. However, this behavior was not observed in this study. A plausible explanation for this discrepancy is that the effect of GCCN on *N*_d_ is highly dependent on conditions like LWC and *N*_d_ itself (e.g., Feingold et al., 1999) and that this study did not capture the appropriate conditions to observe this effect. However, McCoy et al. (2017) did observe a negative coefficient for sea salt and ascribed it to a simulation artifact caused by the intimate link between sea salt generation and wind speed (i.e., turbulence). An attempt to isolate the effects of sea salt and turbulence on *N*_d_ is provided in Sect. 3.1.1.

Menon et al. (2002) and McCoy et al. (2017, 2018) are among the few studies that have used multiple species to predict *N*_d_ (Table 7). Menon et al. (2002) used three species (sulfate, organic matter, and sea salt). McCoy et al. (2017, 2018) used five species (sulfate, sea salt, black carbon, organic carbon, and dust), but the 2017 study found the contribution of organic matter to be negligible. In order to intercompare results with previous studies, we selected species homologous to those of McCoy et al. (2017, 2018). We select ${\text{NSS-SO}}_{4}^{2-}$ for sulfate, Na for sea salt, oxalate for organic carbon, and Fe for dust. We did not measure a species analogous to black carbon. The subsequent analysis examines only these four species using single-predictor regressions.

### 3.3 Analysis of meteorological factors through binning

Historically, the effect that meteorological factors have on the composition-*N*_d_ (or composition-*N*_CCN_) empirical relationship has been examined by analyzing regressions after binning by turbulence (Leaitch et al., 1996), cloud type (Leaitch et al., 1992; Novakov and Penner, 1993), and region (McCoy et al., 2018). The following sections address the effects of turbulence, smoke influence, and location along cloud depth.

#### 3.3.1 Effect of turbulence

**Figure 5.**
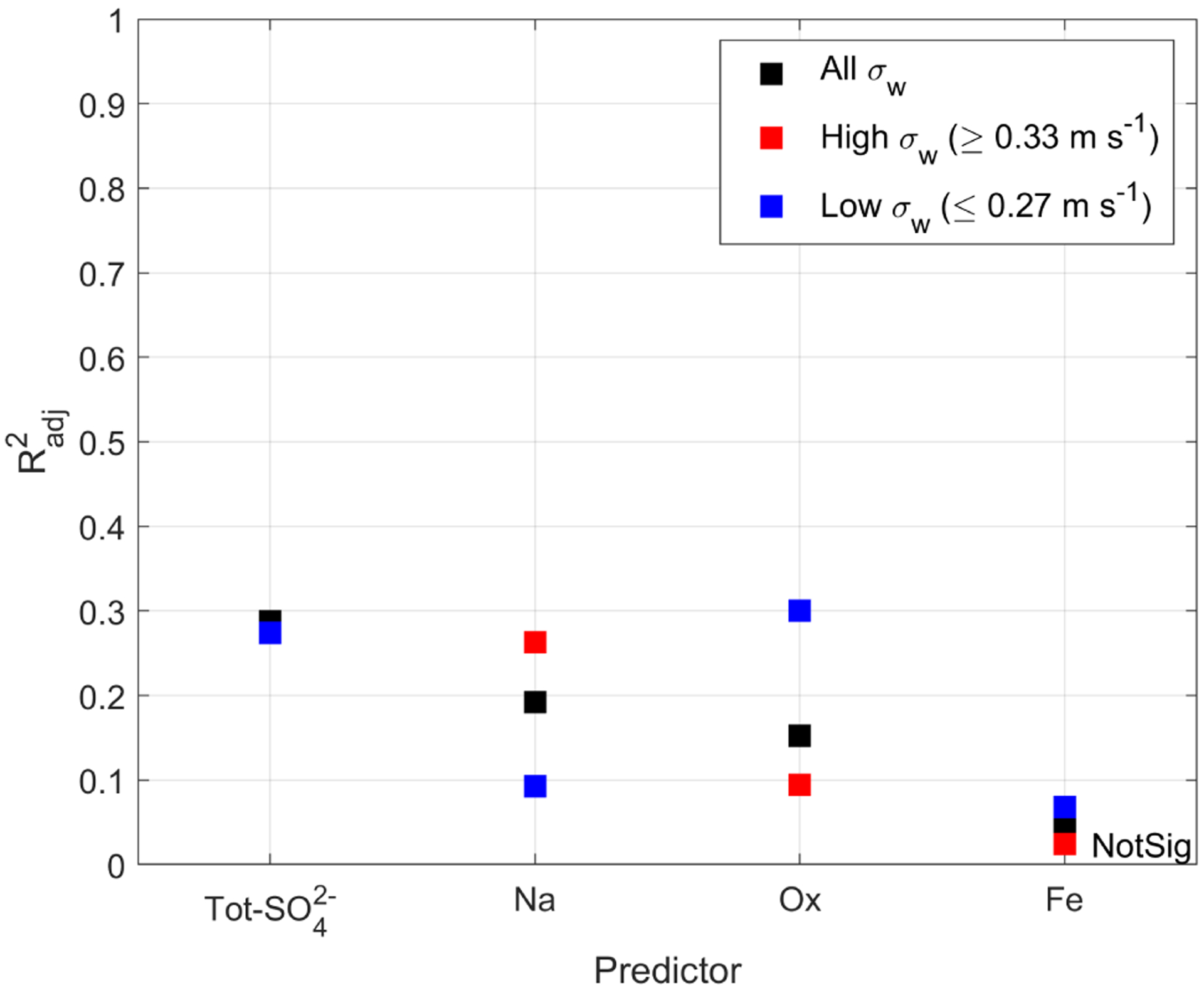
Effect of turbulence (quantified using *σ*_*w*_) on the ability of a single species to predict *N*_d_. For ${\text{NSS-SO}}_{4}^{2-}$; the high (red) and low (blue) *σ*_*w*_ data points overlap. NotSig represents not statistically significant according to the definition in Sect. 2.5.

Building upon the work of Leaitch et al. (1996), who studied how turbulence affects the correlation between ${\text{Tot-SO}}_{4}^{2-}$ and *N*_d_, this study extends that analysis to examine four additional species. Similar to Leaitch et al. (1996), this study quantified turbulence by the standard deviation of vertical wind speed (*σ*_*w*_). Our range of *σ*_*w*_ was 0.10–0.51 ms^−1^. Low turbulence was considered to be in the bottom 33rd percentile (≤ 0.27 ms^−1^), whereas high turbulence was taken to be values in the top 33rd percentile (≥ 0.33 ms^−1^). Leaitch el al. (1996) considered low and high turbulence to be *σ*_*w*_ < 0.17 ms^−1^ and *σ*_*w*_ > 0.23 ms^−1^, respectively, and it is worth noting that only 5 of our 385 samples are considered low turbulence according to the criterion of Leaitch et al. (1996). Figure 5 and Table 8 show how $R_{\text{adj}}^{2}$ depends on the predicting species and the turbulence regime; the scatterplots from which the $R_{\text{adj}}^{2}$ values are taken are shown in Fig. S3.

For ${\text{NSS-SO}}_{4}^{2-}$, there is no significant difference in $R_{\text{adj}}^{2}$ when comparing all the points or by binning by *σ*_*w*_. However, this is not the case for ${\text{Tot-SO}}_{4}^{2-}$, in which there is a large difference in the degree of correlation ($R_{\text{adj}}^{2}=0.27$ and $R_{\text{adj}}^{2}=0.55$ for low *σ*_*w*_ and high *σ*_*w*_, respectively). This is in agreement with Leaitch et al. (1996), in which the correlation (albeit, not log-log) between ${\text{Tot-SO}}_{4}^{2-}$ and *N*_d_ yielded an *R*^2^ = 0.53 and *R*^2^ = 0.91 for low and high *σ*_*w*_, respectively. The difference in the behavior between ${\text{Tot-SO}}_{4}^{2-}$ and ${\text{NSS-SO}}_{4}^{2-}$ hints that the sea salt contributions to ${\text{SO}}_{4}^{2-}$ (i.e., ocean-derived species) are the ones affected by turbulence and hence explains the insensitivity that ${\text{NSS-SO}}_{4}^{2-}$ has to turbulence.

For Ox, the correlation improves at low turbulence ($R_{\text{adj}}^{2}=0.30$) but not at high turbulence ($R_{\text{adj}}^{2}=0.09$). We believe Ox behaves differently than Na, because it does not necessarily just enter the cloud from below via updrafts but rather it enters the cloud from above via entrainment of air from the free troposphere that can at times be enriched with organic species in the study region (Coggon et al., 2014; Crosbie et al., 2016; Hersey et al., 2009; Sorooshian et al., 2007). For Fe, all turbulence scenarios yield a low correlation between Fe and *N*_d_, indicating that, overall, Fe is not a good predictor for *N*_d_.

**Figure 6.**
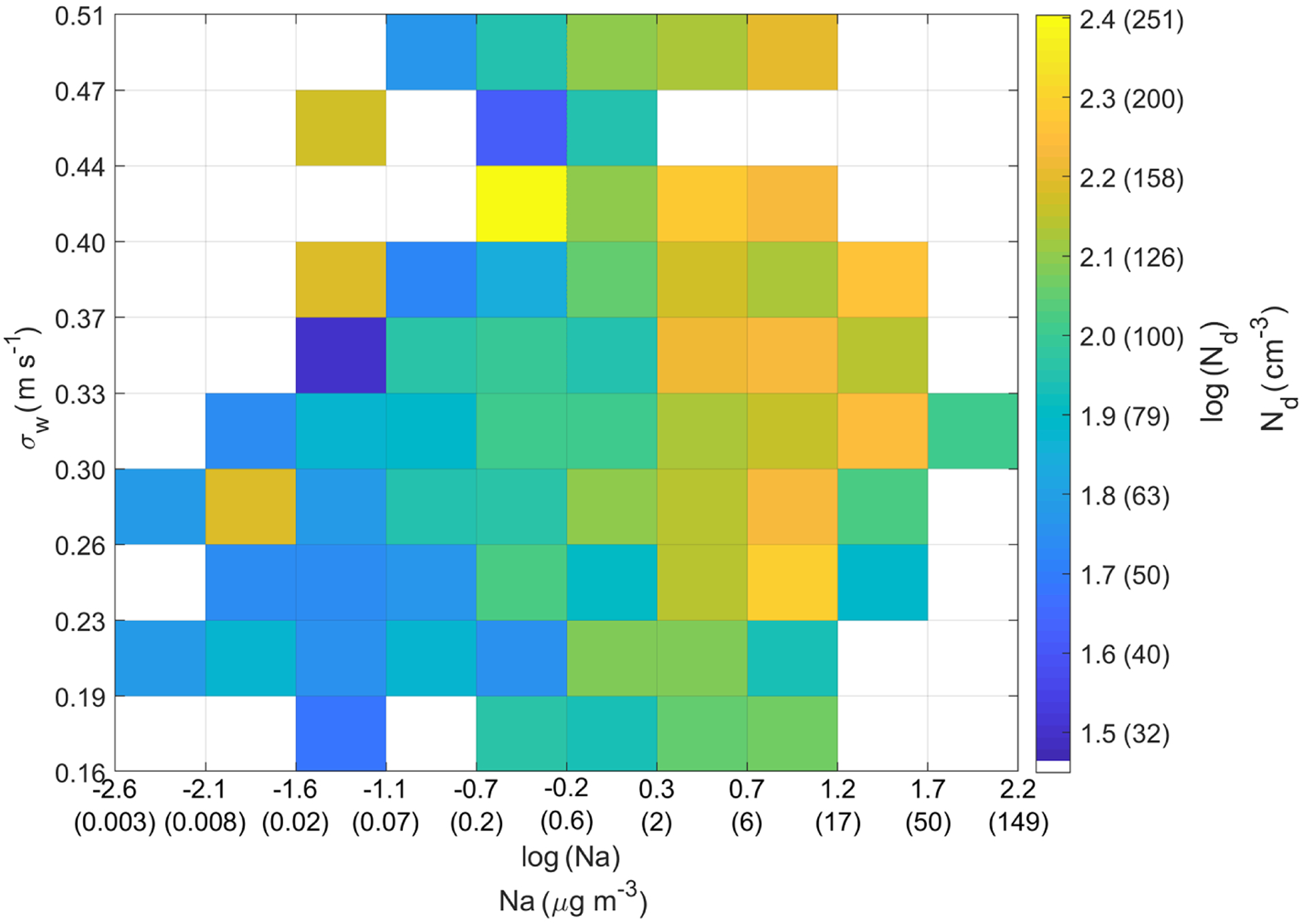
Heatmap showing the dependence of *N*_d_ on both *σ*_*w*_ and Na. The lower and upper bounds for the *x* axis, *y* axis, and color bar cover the entire range of *σ*_*w*_, Na, and *N*_d_, respectively. To assist in physical interpretation, the tick markings on the *x* axis and color bar show two numbers: those without parenthesis correspond to log(Na) or log(*N*_d_) and those within parenthesis correspond to Na or *N*_d_, in their respective units.

For Na, there is a better correlation at high turbulent conditions than at smooth conditions ($R_{\text{adj}}^{2}=0.26$ and $R_{\text{adj}}^{2}=0.09$ for high and low *σ*_*w*_, respectively). This further strengthens the argument that turbulence plays an important role in the vertical transport of sea salt (and other ocean emissions) from the ocean surface to the cloud base. The present data set allows for deeper analysis into the entangled effects of sea salt and turbulence on *N*_d_. More specifically, aerosol reanalysis products like those from MERRA-2 calculate the mass concentration of sea salt via parameterizations that link wind speed to sea salt emissions (Gong et al., 2003; Randles et al., 2017). Since wind speed affects turbulence, it follows that sea salt concentrations are not independent from turbulence, as turbulence is used to calculate sea salt concentrations. Subsequently, these sea salt concentrations are used to predict *N*_d_ (e.g., McCoy et al., 2017, 2018). The present study measured both sea salt (quantified by Na) and turbulence (quantified by *σ*_*w*_) and thus offers an opportunity to try to isolate the effects of both factors on *N*_d_ (Fig. 6). Two results emerge. First, more turbulence is correlated to more sea salt, which is consistent with what the models predict (Randles et al., 2017). Second, at a fixed concentration of Na, *N*_d_ does not vary significantly with *σ*_*w*_, as evidenced by a weak change in color. However, at a fixed value of *σ*_*w*_, *N*_d_ does vary significantly with Na, as evidenced by the noticeable change in color. Therefore, the independent measurement of both variables reveals that *N*_d_ is more sensitive to changes in Na than to changes in *σ*_*w*_. We caution that *σ*_*w*_ is not obtained from below the cloud but from within the cloud during sampling time (Fig. S1).

#### 3.3.2 Effect of smoke influence

**Figure 7.**
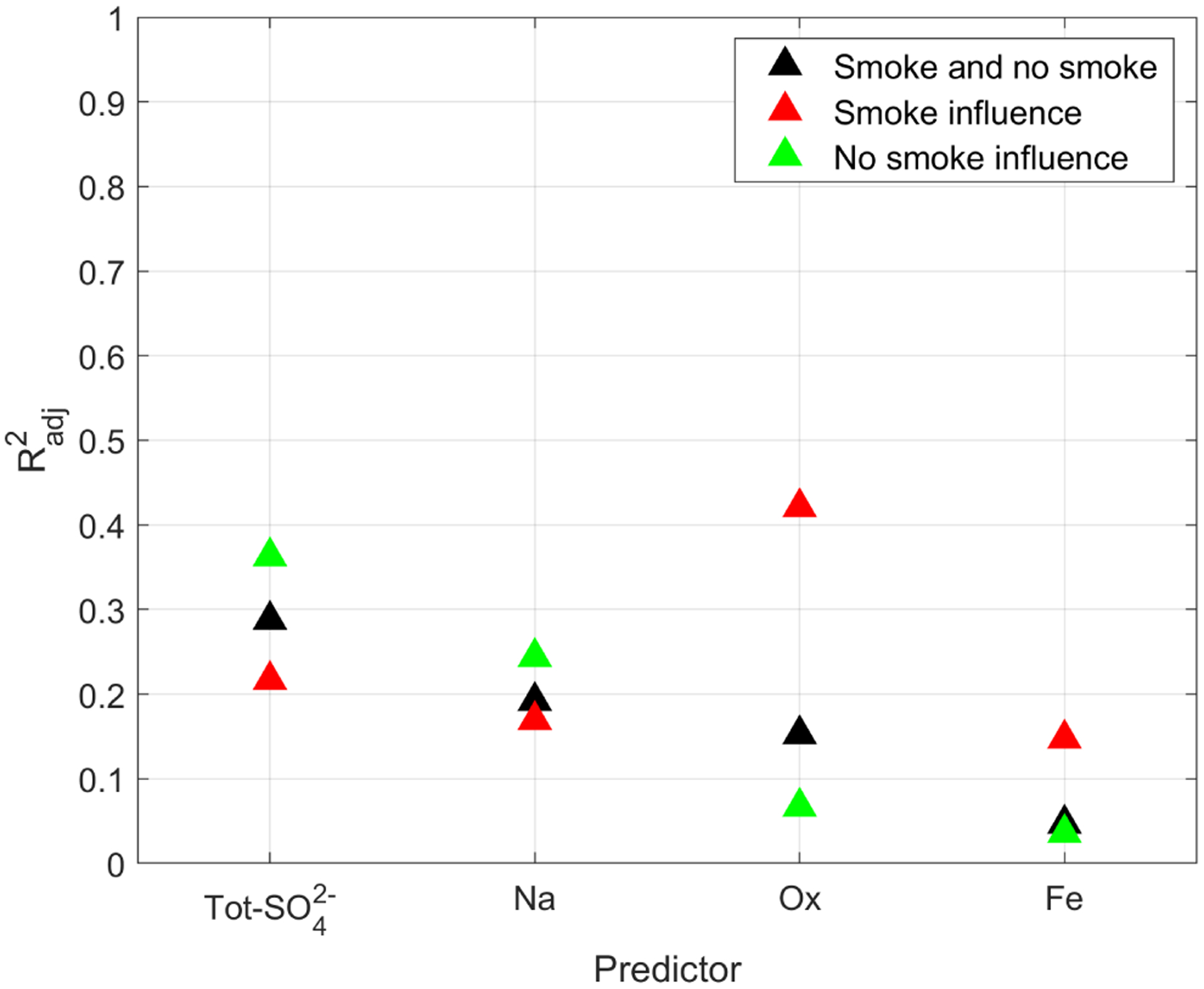
Effect of the influence of smoke on the ability of a single species to predict *N*_d_.

The clouds in the study region are affected by the smoke from wildfires (e.g., Dadashazar et al., 2019; Maudlin et al., 2015; Schlosser et al., 2017). As mentioned in Sect. 2.7, Mardi et al. (2018) used the same data set as this study and identified research flights (RFs) that contained smoke-influenced cloud soundings, namely, NiCE RFs 16–23 and FASE RFs 3–11 and 13–15. In this study, we considered that all cloud water samples collected during the aforementioned RFs were influenced by smoke. Furthermore, we did not distinguish if the smoke was above or below the cloud; this is an important caveat, as cloud microphysical properties seem to depend on the surrounding smoke vertical profile (e.g., Diamond et al, 2018; Koch and Del Genio, 2010). The correlation between *N*_d_ and composition as a function of smoke influence is shown in Fig. 7 and Table 8, and the scatterplots from which the $R_{\text{adj}}^{2}$ values are taken are shown in Fig. S4. Species that are produced during wildfires exhibited an improvement in $R_{\text{adj}}^{2}$ when considering only the smoke-influenced cases. The opposite is true for species not produced during wildfires. More specifically, Ox and Fe showed an increase in correlation for smoke-influenced conditions ($R_{\text{adj}}^{2}=0.42$ and $R_{\text{adj}}^{2}=0.15$ for Ox and Fe, respectively) and a small decrease for smoke-free conditions ($R_{\text{adj}}^{2}=0.07$ and $R_{\text{adj}}^{2}=0.04$ for Ox and Fe, respectively). This is most likely because Ox and Fe concentrations increase during wildfires (e.g., Maudlin et al., 2015) and thus contribute appreciably to the regional CCN during the summertime when wildfires are prevalent.

${\text{NSS-SO}}_{4}^{2-}$ and Na showed a decrease in correlation for smoke-influenced conditions ($R_{\text{adj}}^{2}=0.22$ and $R_{\text{adj}}^{2}=0.17$ for ${\text{NSS-SO}}_{4}^{2-}$ and Na, respectively) and an increase for smoke-free conditions ($R_{\text{adj}}^{2}=0.36$ and $R_{\text{adj}}^{2}=0.24$ for ${\text{NSS-SO}}_{4}^{2-}$ and Na, respectively). We suspect this is because even though wildfires can produce ${\text{NSS-SO}}_{4}^{2-}$ (e.g., Reid et al., 1998) and Na (e.g., Hudson et al., 2004; Silva et al., 1999), these species are not produced as effectively as Ox or Fe. For example, Maudlin et al. (2015) measured aerosol mass concentration in the study region during both smoke-influenced and non-smoke-influenced conditions. They reported an increase in mass concentration for ${\text{NSS-SO}}_{4}^{2-}$, Na, Ox, and Fe to be 30%, 120%, 220%, and 408%, respectively, for submicron particles, and −2%, −28%, 164%, and 97 %, respectively, for supermicrometer particles. Consequently, Ox and Fe are produced more in wildfires in the study region than ${\text{NSS-SO}}_{4}^{2-}$ and Na.

The NiCE (2015) and FASE (2016) campaigns were influenced by smoke originating from different sources. NiCE was influenced by the Big Windy, Whiskey Complex, and Douglas Complex forest fires near the California-Oregon border, with a transport time of approximately 2 d to reach the base of aircraft operations in Marina and adjacent areas where most samples were collected (Maudlin et al., 2015). In contrast, FASE was influenced by the Soberanes Fire approximately 30 km southwest of the aircraft hangar (Braun et al., 2017). Hence, analyzing each campaign separately may provide some insights into the sensitivity of *N*_d_ to smoke from both different fuel types and with varying transport trajectories. NiCE fire data were linked to timber, grass, and shrub models, whereas those from FASE were associated with chaparral, tall grass, and timber (Braun et al., 2017; Mardi et al., 2018). The results are shown in Table 8 and Fig. S4. When comparing FASE to both campaigns combined, the prediction of *N*_d_ using ${\text{NSS-SO}}_{4}^{2-}$, Na, Ox, and Fe is not improved, resulting in $\Delta R_{\text{adj}}^{2}$ values of −0.04, −0.04, 0.01, and −0.03, respectively. However, when comparing NiCE to both campaigns combined, the prediction of *N*_d_ using ${\text{NSS-SO}}_{4}^{2-}$, Na, Ox, and Fe is significantly improved, resulting in $\Delta R_{\text{adj}}^{2}$ values of 0.14, 0.29, 0.18, and 0.13, respectively. The difference between NiCE and FASE could be because different forest fires produce aerosols with varying aerosol chemical signatures and size distributions, as studies in the region have shown (Ma et al., 2019; Mardi et al., 2019). Alternatively, the difference could be due to the small sample size of NiCE (31 samples) as compared to FASE (136 samples) (Table 1). Certainly more research, including larger data sets, is warranted to investigate how different fuel types and plume aging times impact aerosol-cloud interactions.

#### 3.3.3 Effect of in-cloud height

**Figure 8.**
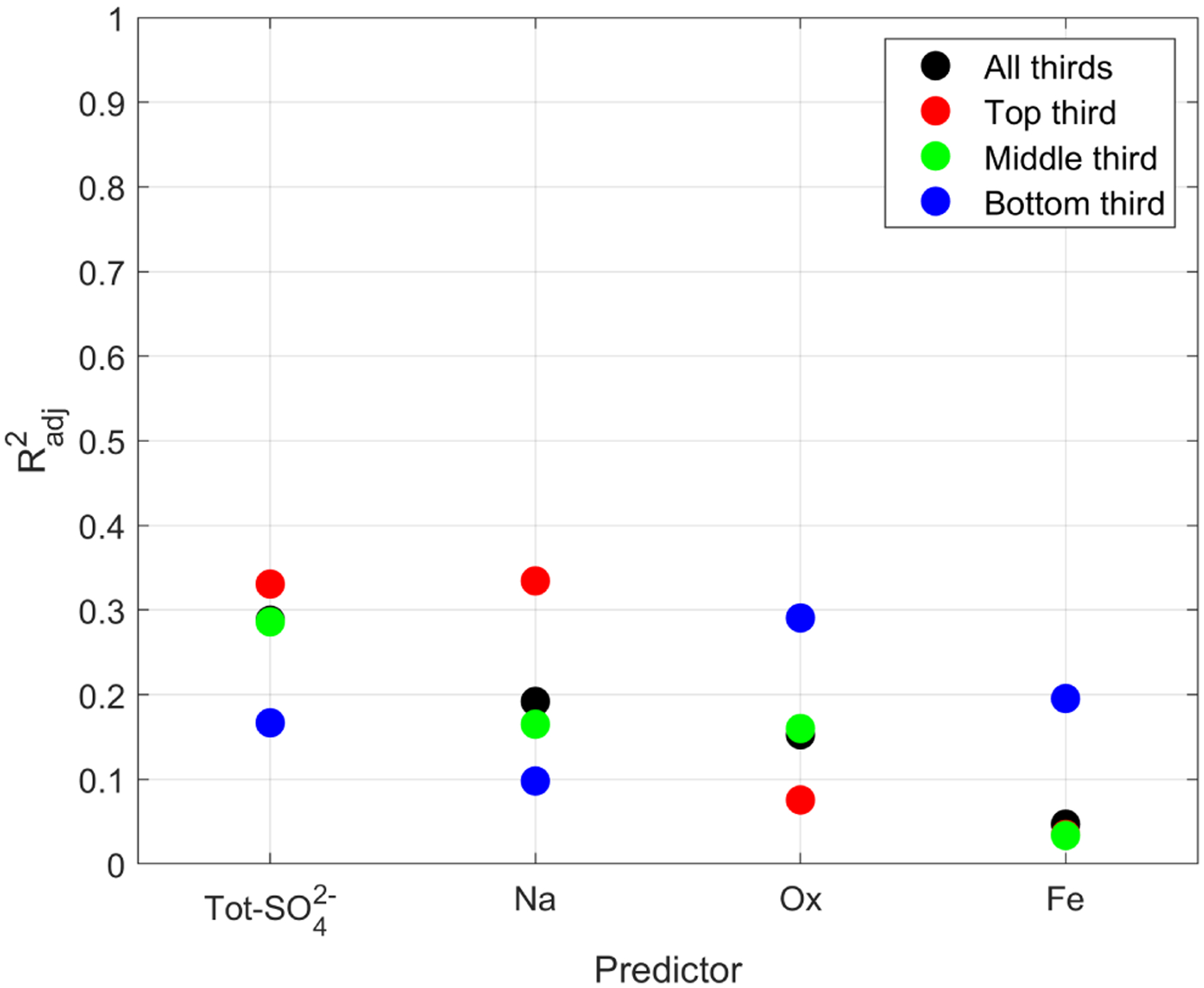
Effect of the influence of normalized cloud height on the ability of a single species to predict *N*_d_. For Fe, the top third of cloud (red) data point overlaps with the middle and bottom thirds of cloud (green and blue) data points.

MacDonald et al. (2018) used the same data set as this study to show that the chemical composition of cloud water varies with height within a cloud. It is therefore reasonable that the *N*_d_-chemical-composition relationship also varies with in-cloud height. The correlation between *N*_d_ and composition as a dependence of in-cloud height is shown in Fig. 8 and Table 8, and the scatterplots from which the $R_{\text{adj}}^{2}$ are taken are shown in Fig. S5.

Ox and Fe exhibit a better correlation when focusing on the bottom third of the cloud ($R_{\text{adj}}^{2}=0.29$ and $R_{\text{adj}}^{2}=0.20$ for Ox and Fe, respectively). When focusing on the top third of the cloud, the correlation decreased for Ox ($R_{\text{adj}}^{2}=0.08$) and remained unchanged for Fe ($R_{\text{adj}}^{2}=0.03$). One possible hypothesis to explain why Ox and Fe are better predictors of *N*_d_ at cloud base is that smokes affects cloud microphysics (*N*_d_ and effective radius) more at cloud base that at cloud top, regardless of whether the smoke was above or below the cloud (Diamond et al., 2018; Mardi et al., 2019).

${\text{NSS-SO}}_{4}^{2-}$ and Na exhibit a better correlation with *N*_d_ when focusing on the top third of the cloud ($R_{\text{adj}}^{2}=0.33$ and $R_{\text{adj}}^{2}=0.33$ for ${\text{NSS-SO}}_{4}^{2-}$ and Na, respectively). The correlation decreases when focusing on the bottom third of the cloud ($R_{\text{adj}}^{2}=0.17$ and $R_{\text{adj}}^{2}=0.10$ for ${\text{NSS-SO}}_{4}^{2-}$ and Na, respectively). ${\text{Tot-SO}}_{4}^{2-}$ also follows this pattern ($R_{\text{adj}}^{2}=0.56$ and $R_{\text{adj}}^{2}=0.22$ for top and bottom, respectively).

It is not entirely clear why ${\text{NSS-SO}}_{4}^{2-}$ and Na would be better correlated with *N*_d_ in the top third of clouds. MacDonald et al. (2018) noted that the concentration of chemical species varies as a function of in-cloud height and is not the same for all species; the concentration of Na is greatest at cloud base, whereas that of ${\text{NSS-SO}}_{4}^{2-}$ and Ox are greatest mid-cloud. It would be expected that the vertical profile of concentration is related to the ability to predict *N*_d_ (i.e., that a larger concentration of a species leads to a better correlation with *N*_d_), but that expectation is not observed in these results. It is also interesting to point out that there is not much difference in $R_{\text{adj}}^{2}$ when considering all cloud thirds versus only the middle third; this makes sense, as almost half of the cloud water samples (46 %) were collected in the middle third of the cloud.

The dependence of the correlation between chemical composition and *N*_d_ on in-cloud height is of relevance to remote sensing, which relies on satellite measurement of cloud-top properties such as cloud-top temperature to then calculate a constant *N*_d_ throughout the cloud depth (e.g., Grosvenor et al., 2018).

## 4 Conclusions

This study used a 4-year data set of airborne measurements collected in warm marine stratocumulus clouds off the California coast and analyzed the extent to which the chemical composition of cloud water can be used to predict *N*_d_. A total of 80 species were filtered to 9 to examine the prediction of *N*_d_ using a single-species model, and then using a multispecies model. The nine species were subsequently filtered to four to examine how the four single-species models were affected by environmental factors, namely, turbulence, smoke influence, and vertical location within a cloud. The most important findings of this paper are the following.

The species that best predicted *N*_d_ is ${\text{Tot-SO}}_{4}^{2-}$ with $R_{\text{adj}}^{2}=0.40$, followed by ${\text{NH}}_{4}^{+}$ ($R_{\text{adj}}^{2}=0.34$), ${\text{NSS-SO}}_{4}^{2-}$ ($R_{\text{adj}}^{2}=0.29$), MSA ($R_{\text{adj}}^{2}=0.26$), and ${\text{NO}}_{3}^{-}$ ($R_{\text{adj}}^{2}=0.24$).

The prediction of *N*_d_ can be improved by using a multispecies model. However, increasing the number of species caused the $R_{\text{adj}}^{2}$ to asymptotically approach ~ 0.6. Furthermore, the regressions with six or more species became statistically insignificant.

Analyzing the three best correlations for each of the *n* species models (where *n* = 1–5) shows that the factors that constitute a good regression are a form of ${\text{SO}}_{4}^{2-}$ (total or non-sea-salt), an ocean emissions tracer, and an organic tracer.

Greater turbulence (approximated as the standard deviation of vertical wind speed) improves the ability of ocean-derived species to predict *N*_d_, as observed when comparing regressions using turbulent data points versus all data points for ${\text{Tot-SO}}_{4}^{2-}$ ($\Delta R_{\text{adj}}^{2}=0.15$) and Na ($\Delta R_{\text{adj}}^{2}=0.07$) but not for ${\text{NSS-SO}}_{4}^{2-}$ ($\Delta R_{\text{adj}}^{2}=-0.01$) or Ox ($\Delta R_{\text{adj}}^{2}=-0.06$).

The influence of smoke significantly affects those species that best predict *N*_d_. Ox (a species known to be produced during biomass burning) was best correlated with *N*_d_ ($R_{\text{adj}}^{2}=0.42$) under smoke-influenced conditions.

Vertical location within the cloud affects the ability to predict *N*_d_. The species that are best correlated with *N*_d_ at cloud top are ${\text{Tot-SO}}_{4}^{2-}$ ($R_{\text{adj}}^{2}=0.56$) and ${\text{NSS-SO}}_{4}^{2-}$ ($R_{\text{adj}}^{2}=0.33$); those best correlated with *N*_d_ at cloud base are fire tracers such as Ox ($R_{\text{adj}}^{2}=0.29$) and Fe ($R_{\text{adj}}^{2}=0.20$), as it has been reported that the base of a cloud is more sensitive to the influence of smoke.

## Supplementary Material

supplement

## Figures and Tables

**Table 1.** Summary of field campaign data sets used in this study and statistics related to cloud water sample collection. Smoke-influenced research flights (RFs) were NiCE RFs 16–23 and FASE RFs 3–11 and 13–15.

Field campaign	Dates (mm/dd/yyyy)	No. of RFs	No. of samples	No. of fire-impacted samples
Eastern Pacific Emitted Aerosol Cloud Experiment (E-PEACE)	07/08/2011–08/18/2011	30	82	0
Nucleation in California Experiment (NiCE)	07/08/2013–08/07/2013	23	119	31
Biological and Oceanic Atmospheric Study (BOAS)	07/02/2015–07/24/2015	15	29	0
Fog and Stratocumulus Evolution experiment (FASE)	07/18/2016–08/12/2016	16	155	136

**Table 2.** Summary of chemical species analyzed in this study. IC = ion chromatography; ICP represents ICP-MS or ICP-QQQ. Note: NSS species, with the exception of ${\text{NSS-SO}}_{4}^{2-}$, were calculated using elements, not ions, hence they have no superscript charge.

Elements (ICP)	
1	Ag
2	Al
3	As
4	B
5	Ba
6	Br
7	C
8	Ca
9	Cd
10	Cl
11	Co
12	Cr
13	Cs
14	Cu
15	Fe
16	Ga
17	Hf
18	I
19	K
20	Li
21	Mg
22	Mn
23	Mo
24	Na
25	Nb
26	Ni
27	P
28	Pb
29	Pd
30	Rb
31	Rh
32	Ru
33	S
34	Sb
35	Se
36	Si
37	Sn
38	Sr
39	Ta
40	Te
41	Ti
42	V
43	W
44	Y
45	Zn
46	Zr
Inorganic ions (IC)	
47	Ammonium (${\text{NH}}_{4}^{+}$)
48	Bromide (Br^−^)
49	Calcium (Ca^2+^)
50	Chloride (Cl^−^)
51	Fluoride (F^−^)
52	Lithium (Li+)
53	Magnesium (Mg^2+^)
54	Methanesulfonic acid (MSA)
55	Nitrate (${\text{NO}}_{3}^{-}$)
56	Nitrite (${\text{NO}}_{2}^{-}$)
57	Potassium (K^+^)
58	Sodium (Na^+^)
59	Sulfate (${\text{SO}}_{4}^{2-}$)
Amines (IC)	
60	Diethylamine (DEA)
61	Dimethylamine (DMA)
NSS species (calculated)	
62	NSS calcium (NSS-Ca)
63	NSS potassium (NSS-K)
64	NSS magnesium (NSS-Mg)
65	NSS sulfate (${\text{NSS-SO}}_{4}^{2-}$)
Organic ions (IC)	
66	Acetate
67	Adipate
68	Butyrate
69	Formate
70	Glutarate
71	Glycolate
72	Glyoxylate
73	Lactate
74	Maleate
75	Malonate
76	Oxalate
77	Propionate
78	Pyruvate
79	Succinate
Acidity (pH)	
80	Hydrogen ion (H^+^)

**Table 3.** Summary of one-predictor models for *N*_d_ based on using any of nine of the final chemical species that were identified after applying the filtering scheme shown in Fig. 2. The coefficients correspond to a linear model of the form log(*N*_d_) = *a*_0_ + *a*_1_ log(*M*_*i*_), where *M*_*i*_ is the mass concentration of species *i*.

		Coefficients	
Species	$R_{\text{adj}}^{2}$	*a*_0_	*a*_1_
${\text{Tot-SO}}_{4}^{2-}$	0.40	2.05	0.32
${\text{NH}}_{4}^{+}$	0.34	2.33	0.25
${\text{NSS-SO}}_{4}^{2-}$	0.29	2.13	0.28
MSA	0.26	2.37	0.31
${\text{NO}}_{3}^{-}$	0.24	2.12	0.25
Na	0.19	2.03	0.13
Ox	0.15	2.26	0.18
V	0.14	2.61	0.15
Fe	0.05	2.26	0.09

**Table 4.** Comparison of coefficient values for studies that correlate *N*_d_ to ${\text{SO}}_{4}^{2-}$ (total or non-sea-salt). The coefficients correspond to a linear model of the form $\text{log}\left(N_{\text{d}}\right)=a_{0}+a_{1}\text{ log}\left({\text{SO}}_{4}^{2-}\right)$.

Reference	*a*_0_	*a*_1_	${\text{SO}}_{4}^{2-}$	*R*^2^	Cloud type
Leaitch et al. (1992)^a^	1.95	0.257	Tot	0.3	Continental stratocumulus
	2.33	0.186	Tot	0.49	Continental cumulus
Novakov et al. (1994)	2.323	0.091	NSS	0.50^b^	Marine stratocumulus
	2.43	−0.056	NSS	0.03	Marine cumulus
Van Dingenen et al. (1995)^c^	2.33	0.4	NSS	0.42	All cloud types combined
Boucher and Lohmann (1995)^c^	2.24	0.257	NSS	^d^	Continental stratus
	2.54	0.186	NSS	^d^	Continental cumulus
	2.06	0.48	NSS	^d^	Marine
	2.21	0.41	NSS	^d^	All cloud types combined
Saxena and Menon (1999)	0.67	0.66	Tot	^d^	Continental orographic clouds
Lowenthal et al. (2004)	2.32	0.74	NSS	0.82	Marine
	2.38	0.49	NSS	0.66	Continental
	2.39	0.5	NSS	0.81	Combined
McCoy et al. (2017)	2.11	0.41	NSS	0.36	Marine stratocumulus

a The units of ${\text{SO}}_{4}^{2-}$ for this regression are nanoequivalents per cubic meter (nEq m^−3^). All other studies report ${\text{SO}}_{4}^{2-}$ in units of micrograms per cubic meter (μg m^−3^). However, the value of the slope (*a*_1_) is not affected by the units of concentration.

b The *R*^2^ has a *p* > 0.05 due to having few data points.

c These regressions were made using data compiled from several studies and assume that *N*_ccn_ ≈ *N*_d_.

d Study does not report *R*^2^.

**Table 5.** The top three statistically significant regressions with the highest $R_{\text{adj}}^{2}$ for a given number of predictors. The coefficients correspond to a linear model of the form log(*N*_d_) = *a*_0_ + Σ*a*_*i*_ log(*P*_*i*_).

	Predictors (*P*_*i*_) and their respective coefficients (*a*_*i*_)											
No. of Predictors	*a*_0_	*a*_1_	*P*_1_	*a*_2_	*P*_2_	*a*_3_	*P*_3_	*a*_4_	*P*_4_	*a*_5_	*P*_5_	$R_{\text{adj}}^{2}$
1	2.05	0.32	${\text{Tot-SO}}_{4}^{2-}$									0.40
	2.33	0.25	${\text{NH}}_{4}^{+}$									0.34
	2.13	0.28	${\text{NSS-SO}}_{4}^{2-}$									0.29
2	2.18	0.22	${\text{Tot-SO}}_{4}^{2-}$	0.12	${\text{NH}}_{4}^{+}$							0.48
	2.43	0.21	MSA	0.15	${\text{NH}}_{4}^{+}$							0.44
	2.25	0.19	${\text{NH}}_{4}^{+}$	0.09	Na							0.42
3	2.25	0.13	${\text{NSS-SO}}_{4}^{2-}$	0.13	${\text{NH}}_{4}^{+}$	0.10	Na					0.50
	2.24	0.19	${\text{Tot-SO}}_{4}^{2-}$	0.10	Ox	0.07	${\text{NH}}_{4}^{+}$					0.49
	2.25	0.17	${\text{Tot-SO}}_{4}^{2-}$	0.11	${\text{NH}}_{4}^{+}$	0.08	MSA					0.49
4	2.32	0.21	${\text{Tot-SO}}_{4}^{2-}$	0.20	Ox	0.09	${\text{NH}}_{4}^{+}$	−0.15	${\text{NO}}_{3}^{-}$			0.52
	2.29	0.11	${\text{NSS-SO}}_{4}^{2-}$	0.10	Ox	0.09	Na	0.08	${\text{NH}}_{4}^{+}$			0.51
	2.31	0.11	${\text{NH}}_{4}^{+}$	0.10	NSS	0.10	MSA	0.08	Na			0.51
5	2.10	0.13	Na	0.12	Ox	0.11	${\text{NSS-SO}}_{4}^{2-}$	0.08	${\text{NH}}_{4}^{+}$	−0.05	V	0.56
	2.40	0.23	Ox	0.13	${\text{NSS-SO}}_{4}^{2-}$	0.10	${\text{NH}}_{4}^{+}$	0.09	Na	−0.17	${\text{NO}}_{3}^{-}$	0.55
	2.36	0.14	${\text{NH}}_{4}^{+}$	0.14	MSA	0.12	${\text{NSS-SO}}_{4}^{2-}$	0.07	Na	−0.08	${\text{NO}}_{3}^{-}$	0.52

**Table 6.** Comparison of regressions containing $\text{NSS}-{\text{SO}}_{4}^{2-}$, Na, and Ox.

	Predictors (*P*_*i*_) and their respective coefficients (*a*_*i*_)							
No. of Predictors	*a*_0_	*a*_1_	*P*_1_	*a*_2_	*P*_2_	*a*_3_	*P*_3_	$R_{\text{adj}}^{2}$
1	2.13	0.28	${\text{NSS-SO}}_{4}^{2-}$					0.29
2	2.12	0.23	${\text{NSS-SO}}_{4}^{2-}$	0.12	Na			0.40
	2.26	0.24	${\text{NSS-SO}}_{4}^{2-}$	0.12	Ox			0.34
3	2.22	0.22	${\text{NSS-SO}}_{4}^{2-}$	0.10	Na	0.08	Ox	0.42

**Table 7.** Results of multivariable regressions from previous studies that have correlated *N*_d_ to mass concentrations. The regression corresponds to a model like Eq. (3). OM represents organic matter, SS represents sea salt, BC represents black carbon, and DU represents dust.

	Predictors (*P*_*i*_) and their respective coefficients (*a*_*i*_)										
Reference	*a*_0_	*a*_1_	*P*_1_	*a*_2_	*P*_2_	*a*_3_	*P*_3_	*a*_4_	*P*_4_	*R*^2^	Cloud type
Menon et al. (2002)*	2.41	0.50	${\text{NSS-SO}}_{4}^{2-}$	0.13	OM						Continental
	2.41	0.50	${\text{NSS-SO}}_{4}^{2-}$	0.13	OM	0.05	SS				Marine
McCoy et al. (2017)	1.78	0.31	${\text{NSS-SO}}_{4}^{2-}$	−0.19	SS	0.057	BC	0.031	DU	0.44	Marine stratocumulus (global average)
McCoy et al. (2018)	2.03	0.2	${\text{NSS-SO}}_{4}^{2-}$	−0.04	SS	−0.03	BC	0	DU	0.08	Marine stratocumulus (just Californian coast)

* This study obtains data from other studies and calculates organic matter.

**Table 8.** Summary of the $R_{\text{adj}}^{2}$ obtained when correlating mass concentration of a species to *N*_d_ under different atmospheric conditions.

		$R_{\text{adj}}^{2}$			
Binning criterion	Data points considered	${\text{NSS-SO}}_{4}^{2-}$	Na	Ox	Fe
None	All	0.29	0.19	0.15	0.05
Turbulence	High *σ*_*w*_	0.27	0.26	0.09	0.02^a^
	Low *σ*_*w*_	0.27	0.09	0.30	0.07
Smoke influence	No smoke	0.36	0.24	0.07	0.04
	Smoke	0.22	0.17	0.42	0.15
	NiCE^b^	0.36	0.46	0.60	0.28
	FASE^b^	0.18	0.13	0.41	0.12
Normalized cloud height	Top third	0.33	0.33	0.08	0.03
	Middle third	0.29	0.16	0.16	0.03
	Bottom third	0.17	0.10	0.29	0.20

a This $R_{\text{adj}}^{2}$ has a *p* value > 0.05.

b Only smoke-influenced samples in this campaign were considered.
