# Supplementary material for: On the relationship between cloud water composition and cloud droplet number concentration
Source: Atmos Chem Phys. Author manuscript; Available in PMC 2020 Dec 2. (PMC7709908; doi:10.5194/acp-20-7645-2020)
Supplement: supplement [file NIHMS1643607-supplement-supplement.pdf]

Supplement of

On the Relationship Between Cloud Water Composition and Cloud Droplet Number Concentration

Alexander B. MacDonald et al.

Correspondence to: armin@email.arizona.edu

Table S1. Limits of detection (LOD) for the species that were measured in this study. IC = Ion Chromatography, ICP = ICP-MS or ICP-QQQ.

Elements (ICP)	LOD (ppt)
Ag	0.74
Al	29.47
As	7.95
B	361.83
Ba	3.70
Br	[a]
C	[a]
Ca	543.10
Cd	4.19
Cl	[a]
Co	0.72
Cr	1.15
Cs	0.73
Cu	1.13
Fe	1.19
Ga	[a]
Hf	0.96
I	[a]
K	10.48
Li	103.65
Mg	14.38
Mn	1.62
Mo	2.26
Na	7.74
Nb	0.52
Ni	2.84
P	770.73
Pb	0.50
Pd	1.68
Rb	1.57
Rh	[a]
Ru	1.44
S	5823.00
Sb	[a]
Se	82.39
Si	126.47
Sn	1.77
Sr	1.10
Ta	0.20
Te	65.46
Ti	39.05
V	1.35
W	[a]
Y	0.5230
Zn	5.8800
Zr	1.0080

Inorganic ions (IC)	LOD (ppm)
Ammonium (NH_4^+)	0.0424
Bromide (Br^-)	0.0251
Calcium (Ca^{2+})	0.0452
Chloride (Cl^-)	0.0021
Fluoride (F^-)	[a]
Lithium (Li^+)	0.0349
Magnesium (Mg^{2+})	0.0369
Methanesulfonic acid (MSA)	0.0123
Nitrate (NO_3^-)	0.0089
Nitrite (NO_2^-)	0.0262
Potassium (K^+)	0.0262
Sodium (Na^+)	0.0435
Sulfate (SO_4^{2-})	0.0120

Organic ions (IC)	LOD (ppm)
Acetate	0.0027
Adipate	0.0227
Butyrate	[a]
Formate	0.0742
Glutarate	0.0063
Glycolate	0.0536
Glyoxylate	0.9448
Lactate	[a]
Maleate	0.0070
Malonate	0.3915
Oxalate	0.0123
Propionate	[a]
Pyruvate	0.0638
Succinate	0.0110

Amines (IC)	LOD (ppm)
Diethylamine (DEA)[b]	0.3152
Dimethylamine (DMA)	0.0527

[a] LODs were not available for these species.

[b] DEA co-elutes with Trimethylamine (TMA), so this LOD is an overestimate.

Table S2. Summary of the number of regressions that were statistically significant in Figure 4. A regression was considered statistical significance if all the p-values for a regression were < 0.05. There is a p-value associated to the overall regression, to each predictor, and to the intercept.

# of predictors	# of regressions	% of regressions that are statistically significant
1	9	100
2	35	66
3	77	22
4	105	10
5	91	8
6	49	0
7	15	0
8	2	0

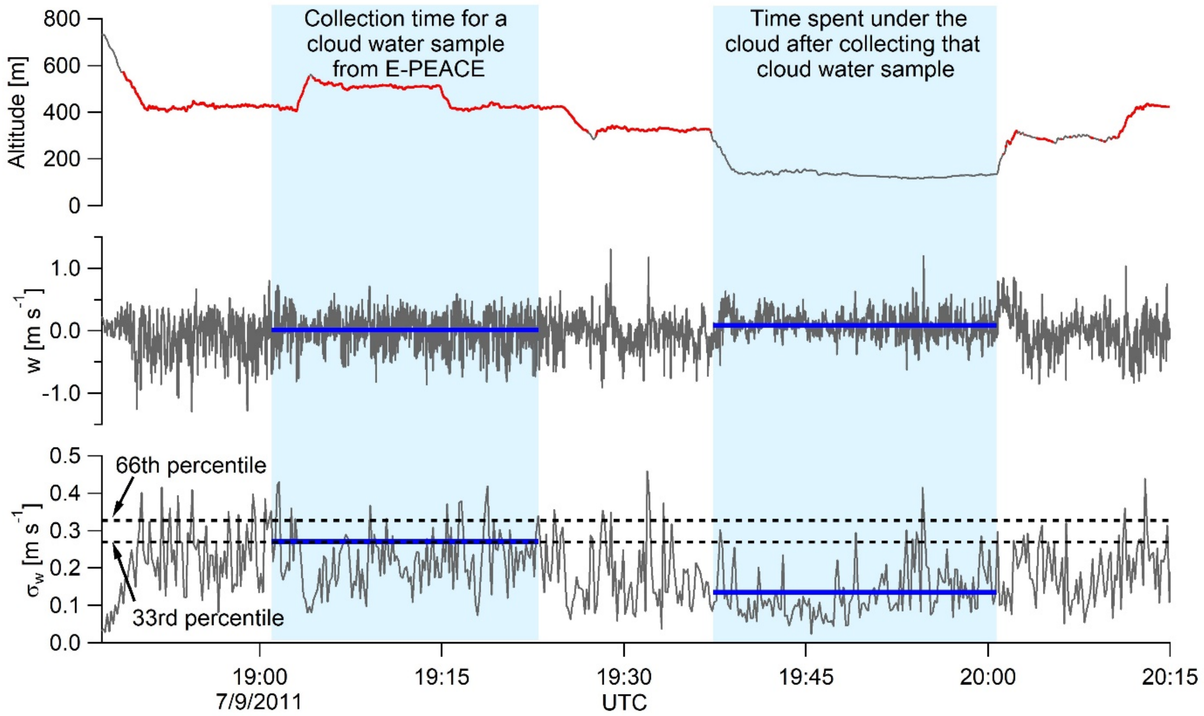

Figure S1. Time series of altitude (top), vertical wind speed (*w*) (middle), and the standard deviation of vertical wind speed (σ_w) (below) for a representative flight on 9 July 2011. The red trace in the top panel indicates when the aircraft was inside the cloud (i.e., LWC $\geq$ 0.02 g m^{-3}). The bold blue lines in the middle and bottom panels are the averages of *w* and σ_w, over the duration of the shaded blue boxes, respectively. The dashed lines in the bottom panel represent the 33rd percentile and 66th percentile of the data in this study.

MSA	1								
NH_4^+	0.36	1							
NO_3^-	0.42	0.48	1						
Ox	0.51	0.55	0.39	1					
Tot-SO_4^{2-}	0.50	0.42	0.43	0.20	1				
NSS-SO_4^{2-}	0.18	0.26	0.36	0.08	0.60	1			
Na	0.35	0.20	0.12	0.13	0.53	0.05	1		
Fe	0.20	0.14	0.22	0.23	0.07	0.02	0.03	1	
V	0.07	0.27	0.49	0.04	0.28	0.48	0.03	0.04	1
	MSA	NH_4^+	NO_3^-	Ox	Tot-SO_4^{2-}	NSS-SO_4^{2-}	Na	Fe	V

Figure S2. Correlation matrix of R^2_{adj} for the nine filtered species used to predict cloud droplet number concentration (N_d). All values are statistically significant (p-value < 0.05). The cells are color coded to highlight low values (red) and high values (green).

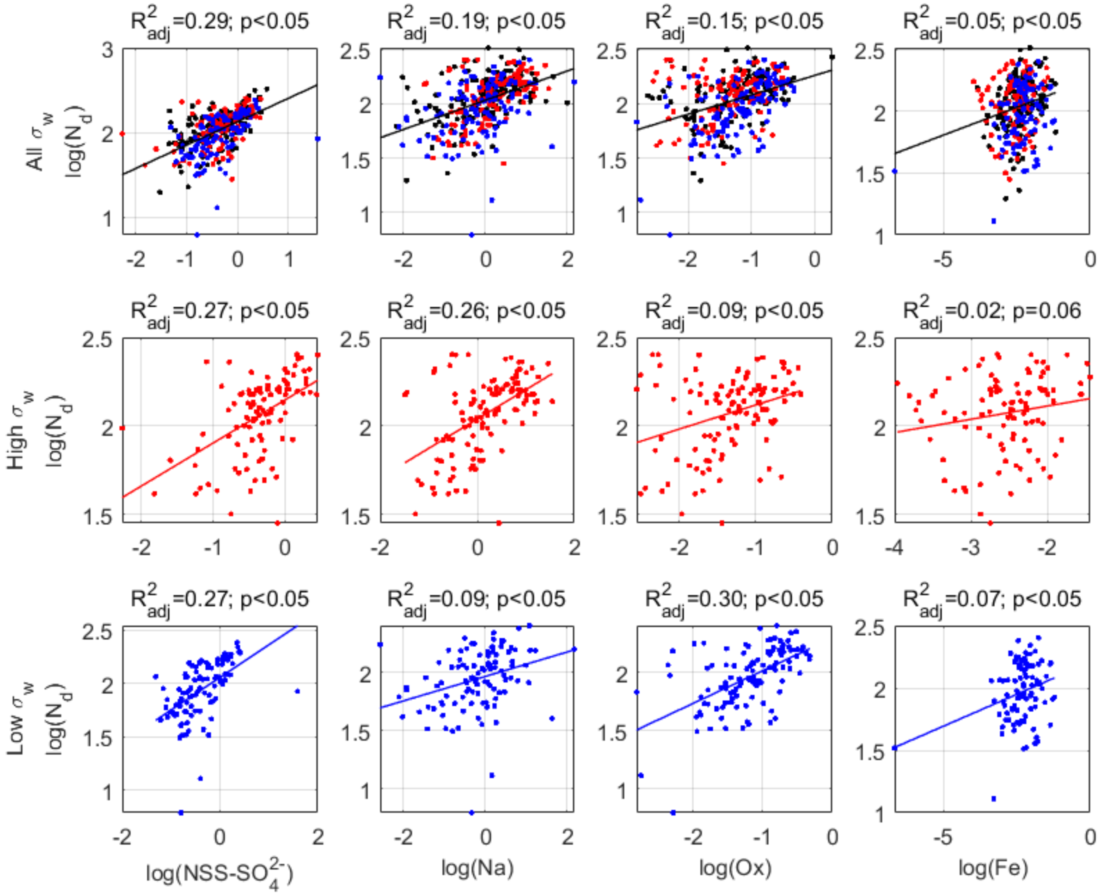

Figure S3. Scatterplots of four selected species when binning by σ_w. These four species were selected owing to their ability to represent distinct aerosol sources in the study region. Red: top 33rd percentile ($\sigma_w \geq 0.33$ m s^{-1}); Blue: bottom 33rd percentile ($\sigma_w \leq 0.27$ m s^{-1}); Black: between bottom and top percentiles (0.27 m s^{-1} $\leq \sigma_w \leq 0.33$ m s^{-1}).

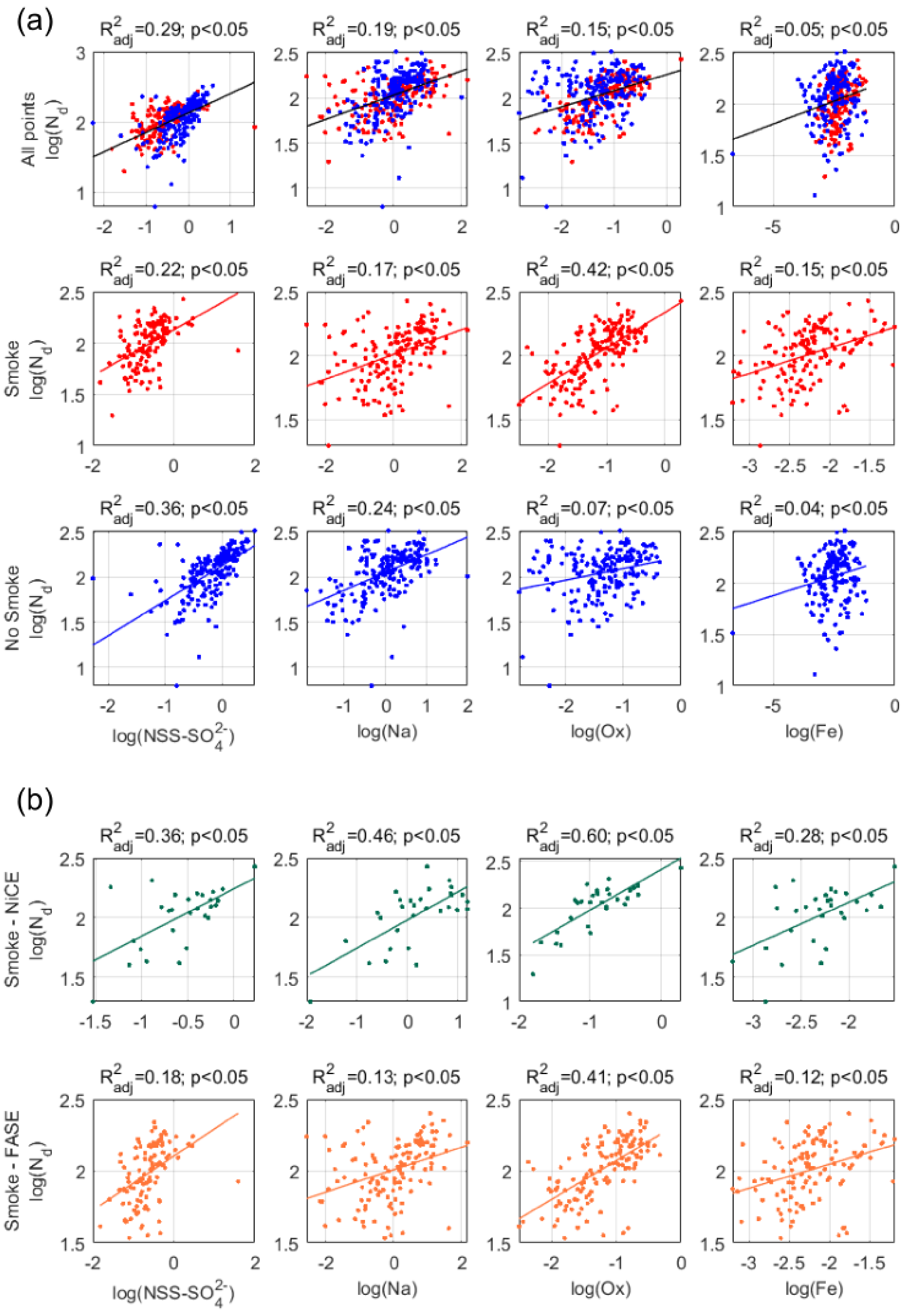

Figure S4. Scatterplots of four selected species when binning by smoke influence. (a) The NiCE (2015) and FASE (2016) campaigns are considered together. Black: Smoke-influence and no-

smoke influence combined; Red: smoke influence; Blue: no smoke influence. (b) The NiCE and FASE campaigns are considered separately. Green: NiCE; Orange: FASE.

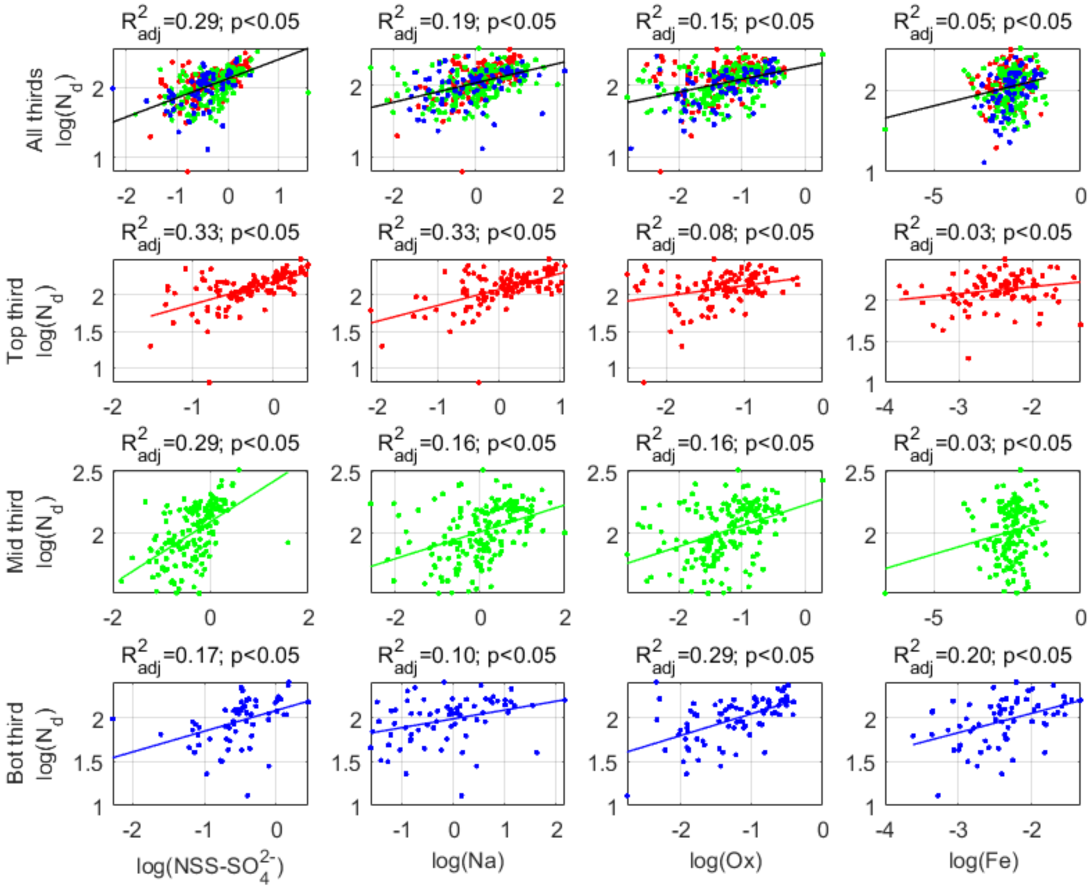

Figure S5. Scatterplots of four selected species when binning by normalized in-cloud height. Red: top third; Green: mid third; Blue: bottom third.
